# Natural deep eutectic solvent ultrasound extraction and imprinting recognition for sustainable harvesting of flavonoid aglycones from safflower: Experimental and multi-scale simulations

**DOI:** 10.1016/j.ultsonch.2026.108128

**Published:** 2026-10-22

**Authors:** Zhiliu Liu, Kechun Wang, Xiaoqing Zheng, Xi Zhao, Yidan Jia, Yan Wang, Beibei Xiang, Lijie Wu, Xiaoxia Li

**Affiliations:** a School of Chinese Materia Medica, Tianjin University of Traditional Chinese Medicine, Tianjin 301617, China; b State Key Laboratory of Chinese Medicine Modernization, Tianjin University of Traditional Chinese Medicine, Tianjin 301617, China; c Tianjin Key Laboratory of Therapeutic Substance of Traditional Chinese Medicine, Tianjin University of Traditional Chinese Medicine, Tianjin 301617, China; d China-Ghana Belt and Road Joint Laboratory on Traditional Medicine, Tianjin University of Traditional Chinese Medicine, Tianjin 301617, China; e School of Chinese Medicine and Pharmaceutical Engineering, Tianjin University of Traditional Chinese Medicine, Tianjin 301617, China

**Keywords:** Safflower, Flavonoid aglycones, Natural deep eutectic solvents, Sonoprocessing, Molecularly imprinted polymers, Multi-scale simulations

## Abstract

Flavonoid aglycones derived from safflower are valuable antioxidants, however, conventional extraction and separation methods rely on large volumes of organic solvents and suffer from poor selectivity. Therefore, this study developed a new integrated strategy that combines ultrasound-assisted natural deep eutectic solvent (NADES) green extraction with molecularly imprinted polymer specific separation, achieving enrichment and specific separation of flavonoid aglycones in safflower. A choline chloride-based NADES was screened as the optimal extractant under ultrasonic assistance (40 kHz, 100 W), with the extraction yield of flavonoid aglycones from safflower reaching as high as 5.37 mg/g. Acoustic cavitation-bubble oscillation, growth, and implosive collapse-generates shear forces, micro-jets, and turbulence that overcome the high viscosity of NADES, facilitating solvent penetration and mass transfer. Density functional theory (DFT) and molecular dynamics (MD) simulations revealed hydrogen bonding and van der Waals interactions as the primary driving forces governing extraction. Guided by DFT-predicted template-to-monomer ratios, a surface molecularly imprinted polymer (SMIP) with specific selectivity for flavonoid aglycones was fabricated on MOF SU-102, exploiting its large surface area and mesoporous structure to overcome the low capacity of traditional MIPs. The resulting SMIP exhibited an adsorption capacity as high as 44.639 mg/g and a maximum imprinting factor of 1.80. Machine learning (ML) was employed to optimize adsorption conditions, reducing experimental effort and cost. The NADES extract was processed through a SMIP-packed syringe-based SPE cartridge, which effectively eliminated interference from complex matrices and efficiently enriched flavonoid aglycones, followed by HPLC detection. The method achieved excellent precision (RSD = 0.318%) and maintained > 90% recovery after seven adsorption–desorption cycles. This NADES-SMIP-SPE platform offers a green, selective, and reusable route for safflower valorization and isolation of bioactive compounds from complex plant matrices. In addition, the green NADES and recyclable, highly selective SMIP can help reduce energy consumption and costs in the industrial separation of plant active ingredients, showing promising potential for scalable and industrial application.

## Graphical abstract

**Figure:**
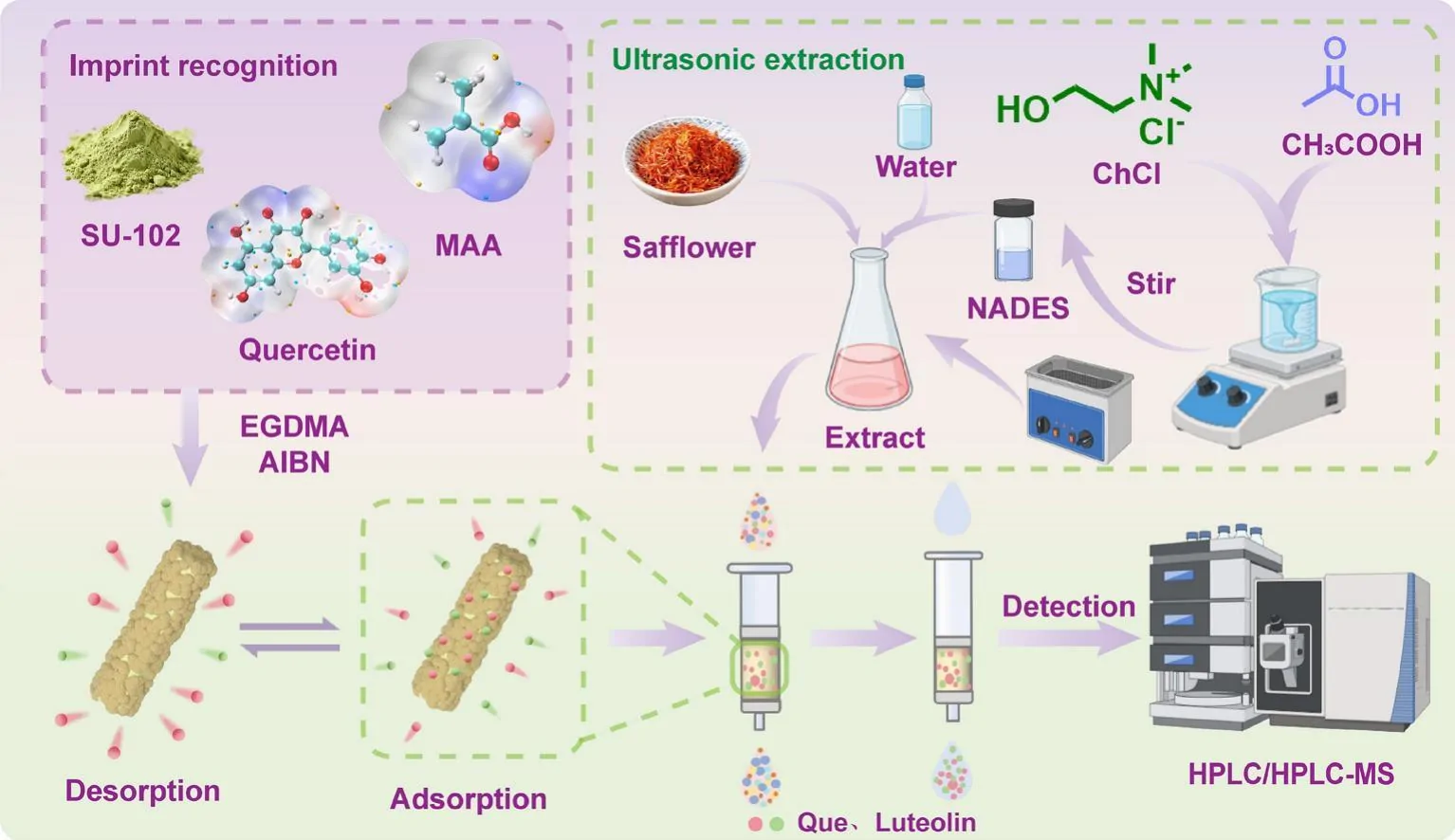

## 1 Introduction

Safflower (*Carthamus tinctorius* L.) is an annual herbaceous plant of the Asteraceae family, widely cultivated for its medicinal and industrial applications. Its primary bioactive constituents-flavonoid aglycones including quercetin, luteolin, kaempferol, and apigenin-have attracted considerable attention due to their potent antioxidant activities, which effectively scavenge free radicals and alleviate oxidative stress-induced cellular damage [1]. Given these health-promoting properties, the efficient and selective extraction and separation of these flavonoid aglycones from safflower is not only a prerequisite for comprehensively elucidating the chemical basis of this medicinal plant, but also a key step in ensuring the reliability of subsequent bioactivity studies and promoting the sustainable utilization of this valuable plant resource [2].

Conventional methods for flavonoid extraction from plant materials, such as maceration, reflux extraction, and Soxhlet extraction, rely on organic solvents. While operationally straightforward, these techniques suffer from long time, high solvent consumption, increased environmental and safety risks, low selectivity, and elevated co-extracted impurities in the final extracts [3], [4]. These limitations have motivated growing interest in developing novel extraction techniques that combine environmental sustainability with high selectivity and efficiency. Among the emerging green solvents, natural deep eutectic solvents (NADES) have proven particularly promising [5]. NADES are stable liquid systems formed through hydrogen-bond interactions between naturally occurring primary metabolites-such as choline chloride, betaine, organic acids, and sugars-mixed at specific molar ratios [6], [7]. NADES offer advantages including simple preparation, low cost, biocompatibility, and environmental friendliness [8], [9].

However, the Achilles' heel of NADES lies in their inherently high viscosity, which severely restricts mass transfer and limits extraction efficiency. This is precisely where ultrasound-assisted extraction (UAE) provides a compelling and tailored solution. UAE is driven by acoustic cavitation-the periodic oscillation, growth, and violent implosive collapse of microbubbles in the liquid medium. This phenomenon generates localized extreme conditions: intense shear forces, vigorous micro-jets, and strong turbulence that physically disrupt plant cell walls and overcome the rheological drawbacks of viscous NADES [10]. They also improve flowability and mass transfer. This synergy enables ultrasound-assisted NADES extraction to achieve high extraction yields, offering a promising route for the green extraction of natural active compounds [11], [12]. A dynamic interaction between cavitation and NADES has been demonstrated in previous studies. Specifically, the extensive hydrogen-bond network of NADES results in high viscosity, which limits mass transfer and may suppress cavitation efficacy [13], [14]. At the same time, high-power ultrasound markedly accelerates reactions in viscous NADES, mainly through internally generated cavitation [15]. Collectively, these findings confirm a bidirectional interaction between cavitation and NADES during ultrasound-assisted extraction. Wang et al. [16] developed a NADES-UAE method for procyanidins from grape pomace and confirmed its scalability. Yang et al. [17] further demonstrated that a hydrophobic DES (DL-menthol:isopropanol) combined with UAE yielded 2.43-fold higher ursolic and oleanolic acids from *Cornus officinalis* than 70% ethanol, with quantum chemical calculations revealing hydrogen bonding and van der Waals interactions as the driving forces. Despite their green nature, however, NADES suffer from poor selectivity, which makes it difficult to eliminate interference from complex matrix components and thus limits their effectiveness for the targeted enrichment of specific bioactive molecules [18].

To overcome the selectivity bottleneck of NADES-based extraction, molecular imprinting technology (MIT) offers a complementary approach. MIT enables the preparation of materials with predetermined recognition and adsorption capabilities by polymerizing target molecules-or their structural analogs-as templates within a polymer matrix [19]. In recent years, MIT has been successfully applied to the separation and analysis of active constituents from medicinal plants, including alkaloids, flavonoids, and saponins. Hou et al. [20] synthesized a multi-template molecularly imprinted polymer and successfully extracted three saponins from purple yam, achieving a maximum adsorption capacity of 74.825 mg/g. However, in conventionally prepared molecularly imprinted polymers (MIPs), the majority of binding sites are embedded within the polymer bulk, limiting their accessibility to target molecules and resulting in poor binding capacity and slow adsorption kinetics. Surface molecular imprinting technology (SMIT) addresses this limitation by immobilizing template-monomer complexes on the surface of a support material, thereby maximizing the availability of imprinted sites [21]. As SMIT has evolved, researchers have leveraged the intrinsic advantages of support materials-such as suitable pore structures and diverse functional groups-to further enhance specific adsorption performance. Metal-organic frameworks (MOFs), a class of porous crystalline materials formed through the self-assembly of metal ions or clusters with organic ligands, are particularly attractive supports due to their high specific surface areas, tunable pore architectures, and strong adsorption capacities [22]. Combining MOFs as supports with surface imprinting provides additional adsorption sites for the resulting polymers, significantly enhancing their adsorption performance. Compared with the organic ligands commonly used in conventional MOFs, SU-102 adopts naturally derived ellagic acid as the organic ligand, offering a new pathway for the green and sustainable synthesis of MOFs. Ellagic acid is a natural polyphenol widely distributed in plants and can be obtained from plant-derived materials such as bark and fruit peels, as well as related processing wastes. It offers advantages including renewability, wide availability, and low cost [23]. However, to date, the integration of plant-based MOFs with surface imprinting for the selective enrichment of flavonoids from NADES extracts remains largely unexplored.

The rational design and optimization of such integrated separation systems can be greatly accelerated by computational approaches. Density functional theory (DFT) allows precise prediction of the optimal template-to-monomer ratio by calculating binding energies and analyzing intermolecular interactions [24]. Molecular dynamics (MD) simulations provide complementary insights by revealing the dynamic behavior, solvation interactions, and structural stability of target molecules in NADES systems at the molecular scale [25]. Fang et al. [26] extracted luteolin from peanut shells and combined DFT with MD simulations to study intermolecular interactions between various solvents and luteolin, demonstrating that hydrogen bonding was the primary driving mechanism. Furthermore, the adsorption performance of imprinted polymers is influenced by multiple experimental factors, including initial concentration, pH, and temperature, and optimizing these conditions through conventional trial-and-error approaches is resource-intensive. Machine learning (ML) offers a powerful alternative for predicting optimal adsorption conditions [27], [28], [29]. Various algorithms-including linear regression, random forest, and neural networks-have been widely applied in materials science and separation technologies [30], [31]. Taoufik et al. [32] employed ML to predict and optimize caffeine adsorption on copper–aluminum layered double hydroxides, achieving a prediction accuracy of 99.9%. Liu et al. [33] used twelve ML models to predict optimal conditions for ammonia nitrogen adsorption by biochar. These successes underscore the potential of ML for streamlining the optimization of complex separation processes.

Despite the individual advances in NADES extraction, molecular imprinting, and computational modeling, an integrated platform that synergistically combines ultrasound cavitation-driven NADES extraction with MOF-supported SMIP-based selective recognition for the recovery of flavonoid aglycones from medicinal plants has not been reported. In this study, we present a novel separation platform-termed ultrasound-NADES-SMIP-SPE-that integrates green NADES extraction with surface molecularly imprinted solid-phase extraction for the highly efficient and selective enrichment of flavonoid aglycones from safflower. Specifically, a series of NADES were designed and screened as green extractants under UAE, and the extraction conditions, including NADES composition, ultrasonication time, and ultrasonication temperature, were systematically optimized; DFT and MD simulations were employed to elucidate the intermolecular driving forces governing extraction performance. Using quercetin as the template and the plant-based MOF SU-102 as the support material, we synthesized a surface molecularly imprinted polymer (SMIP) with high affinity and selectivity toward flavonoids, guided by DFT predictions of the optimal template-to-monomer ratio. The adsorption performance of the SMIP was systematically evaluated through static, dynamic, and selective adsorption experiments, and the key adsorption parameters were optimized using ML modeling. To elucidate the molecular mechanisms underlying the recognition process, electrostatic potential (ESP) distribution and independent gradient model based on Hirshfeld partition (IGMH) analyses were performed to reveal weak intermolecular interactions. Finally, the NADES-SMIP-SPE platform was validated by applying it to real safflower samples, with the enriched flavonoids quantified by HPLC. Through this multi-scale experimental and computational approach, we demonstrate that the ultrasound-NADES-SMIP-SPE strategy not only provides a sustainable, selective, and robust method for the recovery of high-value flavonoids from safflower, but also offers a versatile platform that can be extended to the targeted separation of other bioactive phytochemicals from complex plant matrices. Fig. 1

**Fig. 1:**
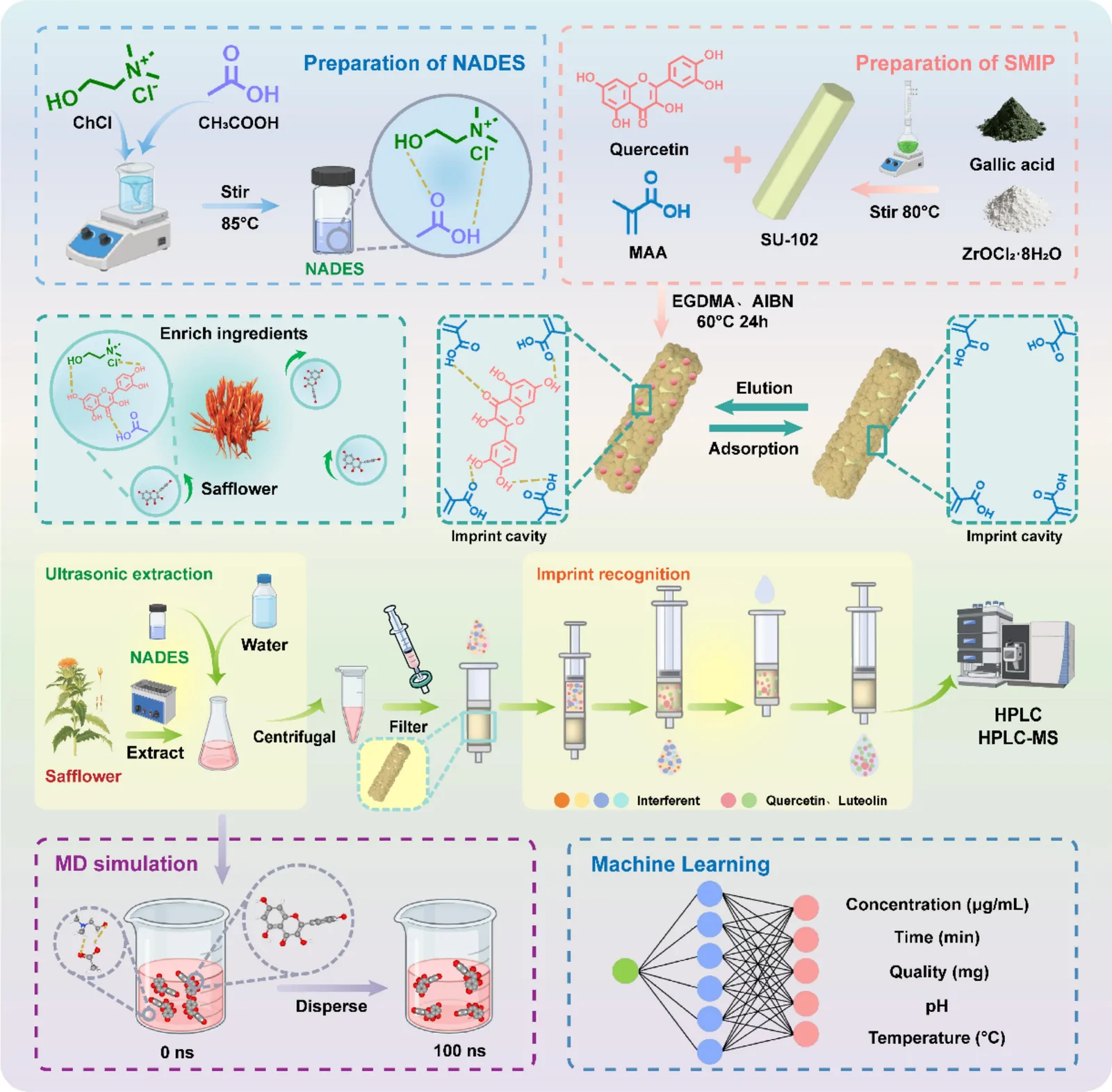
Schematic diagram of NADES-assisted SMIP for the specific separation of flavonoid aglycones from safflower.

## 2 Experiment

### 2.1 Reagents and materials

Quercetin (97%), ellagic acid (98%), ferulic acid (97%), rutin (98%), apigenin (98%), luteolin (98%), kaempferol (98%), methacrylic acid (MAA, 98%), N,N-dimethylformamide (99.5%), 2,2-azobisisobutyronitrile (AIBN, 98%), choline chloride (98%), betaine (98%), D,L-lactic acid (85%～90%), ethylene glycol, glycerol (98%) were purchased from Shanghai Macklin Biochemical Technology Co., Ltd. Zirconium oxychloride octahydrate (98%), ethylene glycol dimethacrylate (EGDMA, 98%), protocatechuic acid (98%), syringic acid (98%) were purchased from Shanghai Aladdin Biochemical Technology Co., Ltd. HPLC-grade acetonitrile (99.9%) and HPLC-grade methanol (99.9%) were purchased from Shanghai Jizhi Biochemical Co., Ltd.

### 2.2 Instruments

Ultrasonic cleaning machine (LABGIC, Hefei Lanjieke Technology Co., Ltd., China). SEM images were acquired using a scanning electron microscope (SU8040, Hitachi). Proton nuclear magnetic resonance (^1^H NMR) spectra were recorded on a JNM-ECZ 600R NMR spectrometer, using D_2_O as the solvent. FTIR spectra were obtained with a PerkinElmer Fourier transform infrared spectrometer (USA). XRD patterns were recorded on an X-ray diffractometer (D8 ADVANCE; Bruker, Germany). Brunauer-Emmett-Teller (BET) analysis was conducted using an automatic specific surface area and pore size analyzer (ASAP 2460, Micromeritics, USA). Thermogravimetric analysis (TGA) was performed on a NETZSCH STA 449 F5/F3 Jupiter instrument (NETZSCH, Germany). XPS spectra were acquired using an X-ray photoelectron spectrometer (Thermo Escalab 250XI, USA). TEM images were acquired using a transmission electron microscope (TECNAI G2 F20, FEI). HPLC analysis was carried out with an Agilent 1260 HPLC-DAD system (Agilent, United States).

### 2.3 Preparation, screening, and optimization of NADES

To establish an environmentally friendly extraction step that maximizes the dissolution of flavonoid aglycones from safflower while minimizing organic solvent consumption, a series of NADES were designed, synthesized, and systematically screened as green extractants.

#### 2.3.1 Preparation of NADES

Betaine and choline chloride were each mixed separately with acetic acid, D, L-lactic acid, glycerol, and ethylene glycol at molar ratios of 1:1, 1:2, 1:3, and 1:4. The mixtures were continuously heated and stirred at 85 °C until homogeneous and stable liquids were formed [34]. They were then allowed to cool and stand at room temperature for subsequent use. Additionally, DFT calculations were employed to investigate the formation process of NADES at the molecular and atomic scales, thereby clarifying its synthesis mechanism.

#### 2.3.2 Screening of NADES Types

Safflower was ground into powder using a high-speed grinder and then filtered through a 60-mesh screen. Nine replicate samples were prepared by accurately weighing 0.250 g of dried safflower powder into separate centrifuge tubes. To each group, 10 mL of 50% aqueous ethanol solution or one of eight different 50% NADES-water mixed solutions (labeled I-IX) was added. The mixtures were ultrasonicated for 30 min (100 W, 40 kHz) and then allowed to stand (The ultrasonic power calibration method can be found in the supplementary materials, and the calibration results are shown in Fig. S1 **and** Table S1). Please refer to the supplementary materials for the specific operation of ultrasonic extraction. The collected supernatants were filtered through a 0.22 μm microporous membrane and then analyzed by HPLC to identify the optimal NADES for extracting compounds from safflower.

HPLC Conditions: Chromatographic column: Hedera ODS-2 C18 column (250 mm × 4.6 mm, 5 μm); Mobile phase: mobile phase A consisted of acetonitrile, 0.2% acetic acid aqueous solution as mobile phase B. Gradient elution program: 0-15 min, 10%∼40% A; 15-20 min, 40%∼70% A; 20-30 min, 70%∼10% A; 30-33 min, maintained at 10% A; Column temperature: 25 °C; Flow rate: 1.0 mL/min; Detection wavelength: 250 nm; Injection volume: 10 μL.

#### 2.3.3 Optimization of NADES concentration

After screening out the optimal NADES, the amount added was optimized. Briefly, 0.250 g of safflower powder was accurately weighed and placed into centrifuge tubes. Then, 10 mL of NADES-water mixtures at concentrations of 10%, 20%, 30%, 40%, and 50% were added, respectively. The mixtures were ultrasonicated for 30 min and allowed to stand. Subsequently, the supernatants were collected, filtered, and injected into HPLC for determination.

#### 2.3.4 Optimization of ultrasound temperature

To investigate the effect of ultrasonic temperature on the extraction yield, five 0.250 g portions of safflower power were accurately weighed and transferred into centrifuge tubes. Subsequently, 10 mL of a NADES-water mixture at an appropriate concentration was added to each tube. The mixtures were subjected to ultrasonic treatment for 30 min at different temperatures (20, 30, 40, 50, and 60 °C) and then allowed to stand. The supernatants were then harvested, passed through a filter, and subjected to HPLC determination.

#### 2.3.5 Optimization of ultrasound time

In a similar vein, ultrasonic duration also constitutes a crucial factor influencing the extraction yield. Following the procedure described above, five safflower samples were weighed and placed into centrifuge tubes, to which 10 mL of NADES solution of appropriate concentration was added. The mixtures were then ultrasonicated for 10, 20, 30, 40, and 50 min, respectively, and thereafter allowed to stand. The supernatants were subsequently collected, filtered, and subjected to HPLC determination.

### 2.4 Synthesis of selective adsorbent (SMIP)

To achieve selective recognition of target flavonoids from the NADES extract, a surface molecularly imprinted polymer (SMIP) was synthesized using quercetin as the template and the plant-based metal-organic framework SU-102 as the support. The template-to-monomer ratio was rationally designed with the assistance of DFT calculations to maximize the formation of specific recognition sites.

#### 2.4.1 Dft-guided design of Template-to-Monomer ratio

Atomic-level adsorption properties was investigated using quantum chemical calculations. At the B3LYP/6-31G(d) level, Gaussian 16W software was employed to carry out geometry optimizations of MAA alone and of quercetin-MAA complexes at various molar ratios. From these results, the most stable configurations of the adsorption systems were identified. In all adsorption models utilized in the simulations, carbon, hydrogen, oxygen, and nitrogen atoms are shown as cyan, white, red, and blue spheres, respectively.

Gaussian 16W together with GaussView 6.0 were utilized for DFT computations in the theoretical simulation part. All structures were subjected to geometry optimization at the B3LYP/6-31G(d) level; prior to optimization, frequency analyses were performed to ensure that each optimized structure resides at an energy minimum on the potential energy surface, indicated by zero imaginary frequencies. The specific color scale range (a.u.) of ESP is shown in Table S2. The ESP distributions of MAA and quercetin, along with atomic charge distributions, were evaluated with Multiwfn 3.8 and VMD 1.9.3. To elucidate inter-fragment interactions, the IGMH was generated using the default isovalue of 0.01. The DFT-D3BJ empirical dispersion correction was adopted throughout the computations to accurately account for weak interactions. Additionally, weak interactions between target molecules and monomers, along with hydrogen-bond donor-acceptor relationships, were investigated via Hirshfeld surface maps and fingerprint plots. The Gibbs free energy and enthalpy of the polymers during the simulation were calculated using the following formulas:

(1) $$\Delta \text{G}={\Delta \text{G}}_{\text{Polymer}}-{\text{G}}_{\text{Target}}-{\text{nG}}_{\text{MAA}}$$

#### 2.4.2 Synthesis of SU-102 support

1.200 g of ellagic acid and 640 mg of zirconium oxychloride octahydrate were weighed and placed into a round-bottom flask, followed by the addition of 40 mL of DMF, 20 mL of deionized water, and 30 mL of acetic acid. The mixture was heated in an oil bath at 80 °C under reflux for 48 h [35]. Subsequently, the resulting suspension was centrifuged at 6000 rpm for 10 min, and the collected green precipitate was washed successively with DMF and deionized water. Finally, the solid product was dried overnight to obtain green SU-102 powder.

#### 2.4.3 Synthesis of SMIP

0.200 g of SU-102 and 1 mmol of quercetin were dissolved in 50 mL of a mixed solvent composed of methanol/acetonitrile (1:1, v/v). Subsequently, 4 mmol of MAA was added, and the mixture was ultrasonicated for 5 min to achieve uniform dispersion. The system was then sealed and stirred at room temperature for 2 h to complete pre-polymerization. After pre-polymerization, 20 mmol of the cross-linker EGDMA and 0.045 g of the initiator AIBN were introduced into the mixture. The solution was degassed by ultrasonication for 20 min and purged with nitrogen flow for 15 min to thoroughly eliminate oxygen. After being sealed, the mixture was allowed to react at 60 °C for 24 h, after which the obtained precipitate was subjected to Soxhlet extraction with a methanol/acetic acid (9:1, v/v) solvent for 48 h until the template molecules were eliminated. The precipitate was further rinsed with methanol to remove residual acetic acid, and the final sample (SMIP) was obtained after drying in an oven at 60 °C for 12 h. The synthesis of SNIP (SU-102 non-imprinted polymer) followed the same procedure as SMIP, except that no template molecule was added. Similarly, the preparation protocols of MIP and NIP were consistent with the above steps, except that SU-102 was not added.

### 2.5 SPE procedure and Analytical methods

To evaluate the selective enrichment performance of the synthesized SMIP and to validate its applicability for real safflower samples, a syringe-based solid-phase extraction protocol was established, coupled with HPLC analysis for quantitative determination of target flavonoids.

#### 2.5.1 Static adsorption experiments

Quercetin methanol solutions were accurately prepared at concentrations of 10, 50, 75, 100, 150, 200, 250, 350, 450, 500, 600, and 650 μg/mL. Then, 5 mg of the adsorbent material was weighed into a 10 mL centrifuge tube, and 5 mL of each quercetin solution at the specified concentrations was added to the respective tubes. Adsorption was carried out by shaking the tubes at room temperature for 6 h. Afterwards, the supernatant was isolated, passed through a 0.22 μm microporous membrane, and injected into HPLC for quercetin analysis. The equilibrium adsorption capacity was calculated using formula (2).

(2) $$Q_{t}=\frac{\left(C_{0}-C_{e}\right)\times V}{m}$$

In addition, the adsorption characteristics of SMIP for the target molecule can be described using the Langmuir and Freundlich models. The static adsorption data were fitted using these models. The fitting equations are shown below:

(3) $$\text{Langmuir model:}\;\;Q_{e}=\frac{Q_{e}K_{L}C_{e}}{1+K_{L}C_{e}}$$

(4) Freundlich model:Qe=KFCe1n

Among these terms, Q_t_ (mg/g) represents the adsorption capacity of the adsorbent for the target molecule; C_0_ (μg/mL) and C_e_ (μg/mL) denote the initial and equilibrium concentrations of the target molecule in the solution, respectively; V (mL) is the volume of the solution added; and m (mg) is the mass of the adsorbent. Q_m_ is the saturated adsorption capacity of the adsorbent; K_L_ and K_F_ are the adsorption equilibrium constants for the Langmuir and Freundlich models, respectively; and Q_e_ is the adsorption capacity at C_e_.

The Sips adsorption model is an empirical isotherm used to describe adsorption behavior on heterogeneous surfaces. It provides a better fit for experimental data than the Langmuir and Freundlich models, particularly when the energy distribution on the adsorbent surface is uneven. The fitting equation for the Sips adsorption model is as follows:

(5) $$Q_{e}=\frac{{Q_{s}\left(K_{s}Ce\right)}^{m}}{{1+(K_{s}Ce)}^{m}}$$

Among these variables, Q_e_ represents the adsorption capacity at equilibrium, Q_s_ denotes the saturated adsorption capacity, K_s_ is the adsorption constant, C_e_ is the equilibrium concentration, and m is a specific factor.

The D-R isotherm is based on Polanyi's adsorption potential theory, and accounts for the heterogeneity of the adsorbent surface as well as the energy changes occurring during the process. The fitting equation is as follows:

(6) $$Q_{e}=Q_{m}exp(-K{Ɛ}^{2})$$

(7) $$E=1/\sqrt{2k}$$

In this equation, K and Ɛ are parameters of the D-R adsorption isotherm. The parameter Ɛ is defined as Ɛ = RT * ln(1 + 1/Ce), where R is the gas constant (8.314 J/mol^-^^1^·K^-^^1^), T (K) is the temperature, C_e_ (mg/L) is the concentration at adsorption equilibrium, Q_e_ (mg/g) is the adsorption equilibrium capacity, Q_m_ is the maximum adsorption capacity, and E represents the adsorption energy.

The Temkin adsorption model is an isotherm model used to describe the effect of interactions between the adsorbate and adsorbent on the heat of adsorption. This model assumes that the heat of adsorption decreases linearly with increasing surface coverage and is applicable to adsorption processes with moderate coverage. The fitting equation for the Temkin adsorption model is as follows:

(8) $$Q_{e}=A+\;B\ln Ce$$

Among them, A and B are the constants of the Temkin isotherm.

#### 2.5.2 Dynamic adsorption experiments

5 mg of adsorbent was accurately weighed into a 10 mL centrifuge tube and 5 mL of quercetin standard solution (500 μg/mL) was added. The mixture was incubated with shaking at room temperature for 1, 5, 10, 15, 30, 60, 120, 180, 240, 300, 360, and 420 min. After incubation, the samples were centrifuged, the supernatant was separated, and the quercetin concentration in the supernatant was measured using HPLC. The adsorption capacity was derived from equation (2). Additionally, the adsorption of the target molecule onto the adsorbent can be characterized using kinetic models. The kinetic adsorption data were fitted using pseudo-first-order and pseudo-second-order models. The formulas are given below

(9) $$\text{Pseudo-first-order kinetic equation:}\;\;ln\left(Q_{e}-Q_{t}\right)=\ln Q_{e}-K_{1}t$$

(10) $$\text{Pseudo-second-order kinetic equation:}\;\;\frac{t}{Q_{t}}=\frac{1}{K_{2}{Q_{e}}^{2}}+\frac{t}{Q_{e}}$$

Here, the pseudo-first-order and pseudo-second-order rate constants are designated as K_1_ and K_2_, respectively; the adsorption capacities at equilibrium and at time t are represented by Q_e_ and Q_t_, respectively, with t being the adsorption time.

#### 2.5.3 Selectivity evaluation

The interfering substances selected for the study were ferulic acid, syringic acid, and protocatechuic acid, all found in safflower. Additionally, compounds structurally similar to apigenin, kaempferol, luteolin, and quercetin were used in selective adsorption experiments. Methanol solutions of these compounds were accurately prepared at a concentration of 500 μg/mL. Then 5 mg each of SMIP and SNIP were weighed and placed into 10 mL centrifuge tubes, and 5 mL of the prepared standard solutions was added. The tubes were shaken at room temperature for 6 h for adsorption, then centrifuged to separate the supernatant. The supernatant was then filtered (0.22 μm) and analyzed by HPLC to determine the compound concentrations. The selectivity factor (SC) was calculated using the following formula:

(11) $$IF=\frac{Q_{\mathrm{MIP}}}{Q_{\mathrm{NIP}}}$$

(12) $$SC=\frac{{\mathrm{IF}}_{M}}{{\mathrm{IF}}_{F}}$$

In Equation (11), IF represents the ratio of molecularly imprinted polymers (MIPs) to non-imprinted polymers (NIPs). In Equation (12), SC denotes the selectivity factor, i.e., the ratio between the imprinting factor for the target molecule and that for other compounds, while IF_M_ and IF_F_ denote the imprinting factors for the template and for other compounds, respectively.

In addition, to further evaluate the practical applicability of SMIP, its competitive adsorption performance was investigated under simulated real-sample conditions. For this purpose, the standard solutions of the above compounds were diluted to prepare a mixed standard solution mimicking safflower extract, with an organic acid concentration of 5 μg/mL and a flavonoid concentration of 50 μg/mL. Subsequently, 5 mg of SMIP was weighed into a centrifuge tube, and 5 mL of the mixed solution was added to examine the competitive adsorption behavior of the compounds. After filtration, the samples were injected into HPLC for detection using the method described in Section 2.3.2.

#### 2.5.4 Effects of Temperature, pH, and adsorbent dosage

To investigate the effect of temperature on the material’s adsorption capacity, a 500 μg/mL quercetin methanol solution was prepared. 5 mg of SMIP was weighed and placed into a 10 mL centrifuge tube, followed by the addition of 5 mL of the solution. Adsorption was conducted by shaking the mixture at 15, 20, 25, 30, 35, and 40 °C for 6 h, after which the samples were centrifuged to obtain the supernatant. The supernatant was filtered (0.22 μm) and subsequently subjected to HPLC analysis for concentration determination.

A 500 μg/mL quercetin solution in methanol was accurately prepared. The pH of the quercetin solution was adjusted to between 3.0 and 10.0 using NaOH (0.01 mol/L) and HCl (0.01 mol/L) standard solutions. After shaking for 6 h at ambient temperature, the solution was centrifuged to obtain the supernatant. The supernatant was filtered through a microporous membrane (0.22 μm) and injected into HPLC for analysis to study the effect of pH on the material's adsorption capacity.

To study the effect of the amount of adsorbent on the adsorption capacity of this material, a 500 μg/mL quercetin methanol solution was prepared. Different amounts of SMIP (2.5, 5, 7.5, 10, 12.5, and 15 mg) were placed in 10 mL centrifuge tubes, followed by the addition of 5 mL solution to each. The samples were shaken for 6 h at room temperature for adsorption to take place, followed by centrifugation to separate the supernatant. After filtering through a microporous membrane (0.22 μm), the solution was injected into HPLC to determine the concentration in the supernatant.

#### 2.5.5 Machine learning for adsorption condition optimization

Machine learning-assisted exploratory analysis of the adsorption behavior of SMIP toward quercetin was carried out using Python 3.13.1. The dataset consisted of 46 sets of adsorption experimental data obtained from static adsorption, dynamic adsorption, pH-effect adsorption, temperature-effect adsorption experiments, and adsorbent mass. Five adsorption variables were selected as input variables, and adsorption capacity was used as the output variable.

Although the dataset size was limited, the adsorption data exhibited univariate nonlinear variation characteristics. Previous studies have shown that when nonlinear relationships exist in small-scale experimental datasets, machine learning methods can serve as an alternative or complementary approach to classical response surface methodology [36], [37]. Therefore, machine learning was introduced in this study as an auxiliary exploratory tool, aiming to summarize the relative influence of each adsorption variable rather than to serve as a standalone predictive optimization model.

In this study, Python 3.13.1 was used to compare the predictive performance of ten models: linear regression, Lasso regression, Ridge regression, decision tree (DT), random forest (RF), AdaBoost, backpropagation neural network (BP), extreme gradient boosting (XGBoost), support vector machine (SVM), and gradient boosting decision tree (GBDT). Considering the limited sample size, the model results were interpreted with caution. The coefficient of determination (R^2^) of the training and test sets was used to evaluate model performance, and the comparison between training and test set performance served only as a preliminary reference for model generalization ability.

The dataset from the single-factor optimization experiment was divided into training and test sets in an 8:2 ratio. The optimal hyperparameters for each model were determined collaboratively using Bayesian optimization combined with grid search cross-validation (CV = 5). Model performance was primarily evaluated using the R^2^ values on both the training and test sets to quantify the models' predictive accuracy. The calculation formula is as follows:

(13) $$R^{2}=1-\sum {\left(yi-xi\right)}^{2}/\sum {\left(yi-\bar{y}i\right)}^{2}\;(i=1-n)$$

Here, *yi* and *xi* represent the predicted and the observed values, respectively. The symbol $\overset{¯}{\mathrm{yi}}$ denotes the mean of the observed values, and n represents the total number of samples.

#### 2.5.6 Reusability and stability Tests

100 mg of the SMIP sample was accurately weighed and placed into the SPE device. Then, 500 μg/mL quercetin methanol solution was injected into the syringe and allowed to slowly flow out through the sieve plate. The remaining liquid was collected, passed through a 0.22 μm microporous filter for later use, and rapid elution of quercetin was carried out with methanol/acetic acid (9:1, v/v) as the eluent. This process was repeated seven times, with pure methanol washing between each cycle to thoroughly remove residual quercetin. The collected solutions were injected into HPLC for detection. The reusability of SMIP was evaluated by calculating the recovery extraction rate of quercetin. Additionally, to study the mechanical stability of the material, the SMIP samples were separately immersed in HCl solution (pH 1), NaOH solution (pH 13), ethyl acetate, methanol, and dichloromethane for 24 h. The resulting samples were then characterized by XRD and FTIR, and the spectra were compared with that of the original SMIP.

#### 2.5.7 HPLC Analytical conditions

HPLC analysis was performed using an Agilent 1260 high-performance liquid chromatograph equipped with a diode array detector. The stationary phase consisted of a Hedera ODS-2 C18 column (4.6 mm × 250 mm, 5 μm). The mobile phase for quercetin analysis consisted of methanol–water (70:30, v/v). The injection volume was 10 μL, the column oven was set to 30 °C, the flow rate was maintained at 1.0 mL/min, and detection was performed at a wavelength of 360 nm. HPLC conditions for other interfering substances are detailed in Table 1. Furthermore, the HPLC-DAD system demonstrated excellent system suitability, with chromatographic peak shapes exhibiting good symmetry (tailing factors close to 1) and theoretical plate counts exceeding 10,000. The RSD values for repeatability and precision were both approximately 1 %. Comprehensive data are summarized in Table S3.

**Table 1:** HPLC conditions of each analyte.

Analyte	Mobile phase	Wavelength (nm)	Column temperature (°C)	Linear range (mg/L)	R^2^
Quercetin	MeOH: H_2_O (v/v 70:30)	360	30	10–700	0.9995
Syringic acid	MeOH: 0.2 % HAc (v/v 50:50)	290	30	100–600	0.9997
Ferulic Acid	MeOH: 0.2 % HAc (v/v 60:40)	320	30	100–600	0.9994
Protocatechuic Acid	MeOH: H_2_O (v/v 35:65)	260	30	100–600	0.9991
Apigenin	MeOH: 0.2 % HAc (v/v 70:30)	340	30	100–600	0.9991
Kaempferol	ACN: 0.2 % HAc (v/v 50:50)	266	30	100–600	0.9996
Luteolin	MeOH: 0.2 % HAc (v/v 70:30)	350	30	100–600	0.9991

#### 2.5.8 Real sample analysis

Based on the screening results from section 2.3, the safflower extract was prepared. After allowing it to stand undisturbed, the extract was filtered through a 0.22 μm microporous membrane. An appropriate amount of the filtrate was taken for mass spectrometry analysis to determine its chemical composition. The remaining extract was then placed in a custom-made simple extraction column and eluted with methanol/acetic acid (9:1, v/v). After collection, the eluate was analyzed by HPLC to evaluate the enrichment efficiency of the SMIP material.

The above safflower extract was analyzed using ultra-high-performance liquid chromatography quadrupole time-of-flight mass spectrometry (UHPLC-QTOF-MS) to identify its components. The specific conditions are described as follows:

Chromatographic conditions: Chromatographic system: UHPLC Agilent 1290 (Agilent Technologies, Waldbronn, Germany); Mass spectrometry analysis system: Agilent 6520-QTOF (Agilent, USA); Chromatographic column: ACQUITY ZORBAX Eclipse XDB-C18 column (2.1 × 150 mm, 1.8 µm); Mobile phase: 0.1% formic acid solution (A) and acetonitrile (B); Corresponding Gradient elution: 0-30 min, 5-95 % (B); Flow rate: 0.3 mL/min; Column temperature: 35 °C; Injection volume: 10.0 μL.

Mass spectrometry conditions (electrospray ionization, ESI): Ionization mode: positive and negative ion modes; Gas flow rate: 11 L/min; Drying gas (N_2_) temperature: 330 °C; Nebulizer pressure: 35 psig; Fragmentor voltage: 150 V; Octopole RF: 750 V; Skimmer voltage: 65 V; Capillary voltage: 4000 V; MS scan range: *m*/*z* 50-1700. MS^2^ parameters: collision energy (CE) set to 10 V and 30 V.

### 2.6 Multi-scale theoretical simulations

To provide a mechanistic understanding of the separation process across different scales-from molecular interactions to macroscopic adsorption behavior-a combination of DFT calculations, MD simulations were employed. These complementary computational approaches reveal the intermolecular forces governing NADES extraction and SMIP recognition, while enabling data-driven optimization of adsorption conditions.

In this study, MD simulations were employed to investigate the extraction effects of different solvents on safflower. The two-dimensional coordinates (SDF format) of quercetin and luteolin were obtained from the PubChem database (https://pubchem.ncbi.nlm.nih.gov/) and submitted to the Automated Topology Builder (ATB) online server (https://atb.uq.edu.au/) to generate three-dimensional coordinates (PDB format) and topology files (ITP format). MD simulations were performed using the GROMACS 2023.51 program with the GROMACS-G54A7FF force field. Ten quercetin or luteolin molecules were randomly placed in a box with dimensions of 10 × 10 × 10 nm, and the solvent environments were set as 50 % ethanol-water, 40 % NADES, and 50 % NADES. Prior to production runs, energy minimization was conducted to eliminate steric clashes and unfavorable contacts. Subsequently, the system was equilibrated for 100 picoseconds (ps) each in the NVT ensemble (constant number of particles, volume, and temperature) and NPT ensemble (constant number of particles, pressure, and temperature) to stabilize temperature and pressure, respectively. These preparation steps ensured that the system was fully relaxed before the molecular dynamics production simulations commenced. Temperature coupling was maintained for all molecular components using the V-rescale thermostat, while pressure control was achieved using the Parrinello-Rahman barostat to ensure system stability. After equilibration, a 100 ns production molecular dynamics simulation was carried out under the NPT ensemble. Subsequently, the trajectory data was analyzed to assess the structural stability of the system and the interaction behavior. Specifically, the trajectory datas were analyzed to assess the structural stability of the system and the interaction behavior, including root mean square deviation (RMSD), hydrogen bond (HB), and solvent-accessible surface area (SASA) analysis using standard GROMACS tools. VMD (Visual Molecular Dynamics) was used to further visualize and examine detailed molecular interactions between molecules.

### 2.7 Method greenness assessment

The environmental friendliness of the present NADES-SMIP-SPE method was evaluated by means of the Analytical GREEnness (AGREE) calculator and the Complex Green Analytical Procedure Index (Complex GAPI) tool, following the respective published procedures. The E-factor was derived from the mass ratio of waste generated to the product recovered.

## 3 Results and Discussion

### 3.1 Green NADES Extraction: Solvent Selection, Optimization, and mechanistic insights

To establish the first module of the integrated separation platform-green extraction-a series of NADES were synthesized, characterized, and screened for their ability to efficiently liberate flavonoid aglycones from safflower biomass. The extraction performance was subsequently rationalized through molecular dynamics simulations, revealing the intermolecular driving forces underlying the observed solvent efficiency.

Appropriate viscosity facilitates efficient extraction of real samples. Excessively high viscosity may cause column blockage and interfere with sample analysis [38], while excessively low viscosity can hinder the elution of chemical components, reducing extraction efficiency [39]. A total of 32 NADES were synthesized (as shown in Fig. 2a), and after preliminary screening, eight were selected for sample extraction, labeled II-IX. Their viscosity and pH data are summarized in Table 2. All eight selected NADES appeared as clear, transparent liquids. To achieve optimal extraction efficiency, the effects of different NADES compositions on the extraction of target components from safflower were first investigated. As shown in Fig. 2b-c, significant differences in extraction efficiency were observed among the various NADES systems. Compared with 50% ethanol, the choline chloride-acetic acid system exhibited the highest extraction yield, reaching 5.37 m g/g, followed by the betaine-acetic acid system; both of which showed significantly higher extraction efficiencies than the other NADES systems. This phenomenon was mainly attributed to the strong hydrogen-bond interactions formed between the hydrogen-bond donors in the NADES and the phenolic hydroxyl groups in the target molecules, thereby enhancing the solubility and mass-transfer efficiency of the target compounds [40], [41]. In addition, the moderate viscosity of this NADES system favored solvent diffusion during the extraction process. Based on these results, choline chloride-acetic acid was selected as the extraction solvent for subsequent experiments.

**Fig. 2:**
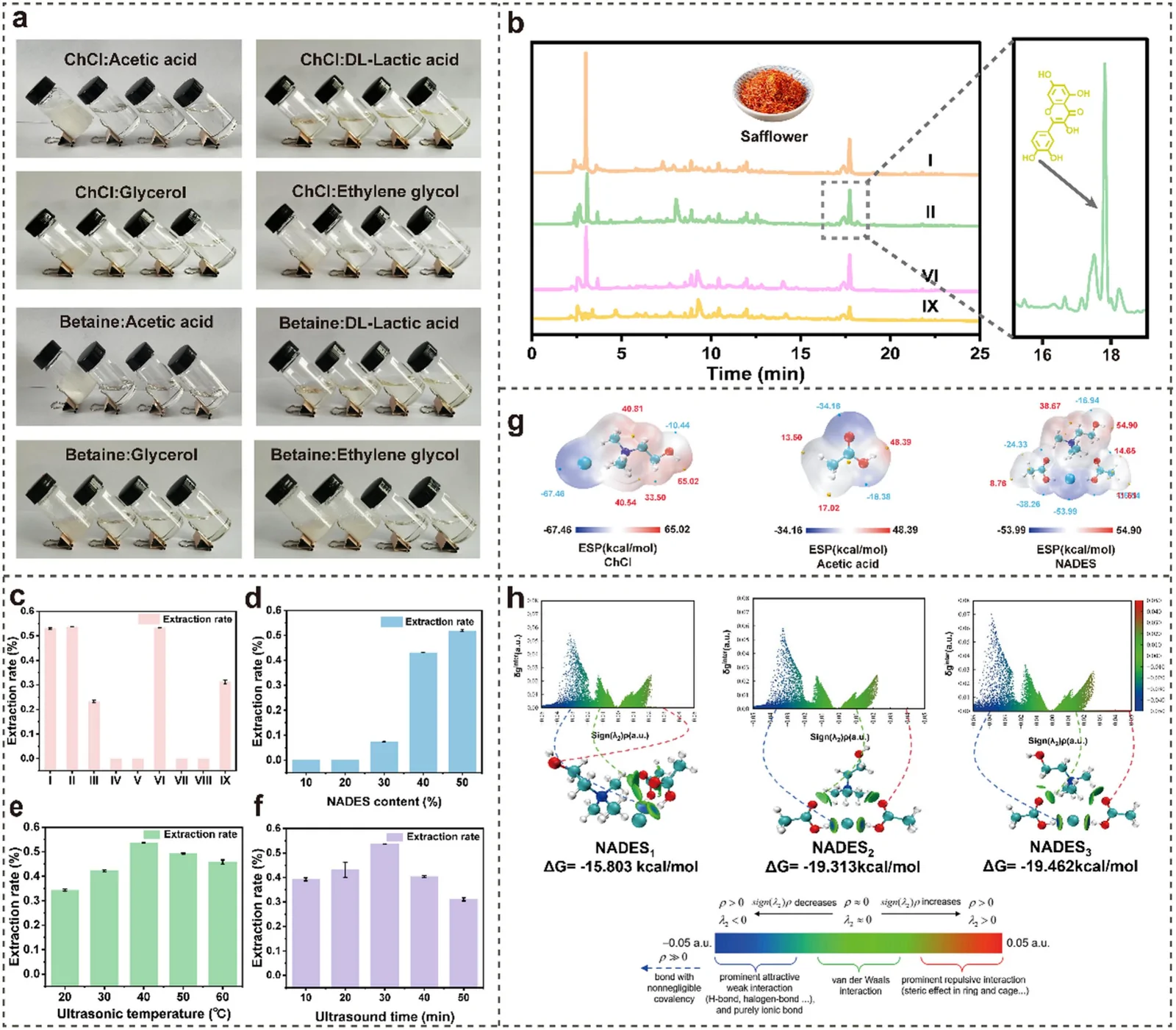
(a) Eight different NADES systems; (b-c) Screening results of different NADES systems; (d) Screening of the optimal NADES ratio; (e) Diagram of the Effect of Ultrasonic Temperature on Extraction Rate; (f) Diagram of the influence of ultrasound time on extraction rate (Unless otherwise stated, all error bars in the figures represent the standard deviation (SD) of three independent experiments (n = 3)); (g) ESP distribution maps of ChCl, acetic acid, and NADES; (h) IGMH scatter plots and rainbow-colored δg^inter^ isosurfaces of the three NADES conformations.

**Table 2:** Details of each NADES (20 °C).

Number	Raw materials	Proportion	Viscosity (mPa·s)	pH
II	ChCl: Acetic acid	1:2	5.1	0.90
III	ChCl: DL-Lactic acid	1:2	13.3	1.72
IV	ChCl: Glycerol	1:2	14.6	5.36
V	ChCl: Ethylene glycol	1:2	3.8	5.06
VI	Betaine: Acetic acid	1:2	30.4	1.37
VII	Betaine: DL-Lactic acid	1:2	15.3	4.02
VIII	Betaine: Glycerol	1:4	46.2	6.63
IX	Betaine: Ethylene glycol	1:3	3.7	6.73

After determining the optimal type of NADES, the effect of NADES content on extraction efficiency was further investigated. As shown in Fig. 2d, the extraction yield increased with increasing NADES content, reaching a maximum when the NADES content was 50%. This improvement can be attributed to the higher NADES concentration in the system, which enhanced the solubility and diffusion rate of the target compounds and strengthened hydrogen-bond interactions between NADES and the target molecules, thereby increasing extraction efficiency. When the NADES concentration exceeds 50%, the viscosity of the aqueous extraction system increases, thereby increasing mass-transfer resistance [42]. High viscosity also makes separation difficult, complicates subsequent filtration, and increases the risks of instrument contamination and chromatographic column clogging [43]. In addition, the experimental results show that the extraction performance differs little within the 40-50% range and further increasing the NADES content does not significantly improve the recovery of the target compounds. Considering extraction efficiency, solution handling, and compatibility with chromatographic analysis, 10-50% was selected as the concentration range for NADES optimization in this study. Therefore, a NADES system containing 50% choline chloride-acetic acid was selected for extraction in subsequent experiments (hereafter referred to as NADES).

Similarly, the effects of ultrasound temperature and time on extraction yield were investigated, as shown in Fig. 2e-f. With increasing ultrasound temperature and time, the extraction yield exhibited a trend of first increasing and then decreasing. This may be attributed to the fact that appropriate temperature and time enhance the ultrasonic cavitation effect, which disrupts the cell walls of safflower and promotes the dissolution of target compounds [44]. However, the already–dissolved components may undergo oxidative degradation at excessively high temperatures or over prolonged durations, ultimately leading to a decline in extraction yield [45]. At higher temperatures, the decrease in flavonoid aglycone extraction yield may be related to the reduced stability of flavonoids under heat treatment and ultrasonic action. Previous studies have shown quercetin may undergo thermal degradation during heating, forming various degradation products through reactions including oxidation, hydroxylation, and cleavage [46]. In addition, ultrasonic treatment can also induce the degradation of flavonoid aglycones through multiple pathways, including oxidation, addition, polymerization, and decomposition [47]. Therefore, under the combined action of temperature and ultrasound, the degradation of flavonoid aglycones results in a decrease in extraction yield.

In addition, the formation mechanism of choline chloride-acetic acid was elucidated using DFT calculations. Fig. 2g displays the ESP distribution maps of choline chloride, acetic acid, and the NADES system, along with schematic diagrams of three different conformations of the optimal NADES (Fig. 2h). Regions colored red correspond to ESP maximum values, while blue corresponds to minimum values. The red zones highlight electrophilic sites that can be attacked by nucleophiles and also serve as hydrogen-bond donor sites. Conversely, the blue regions represent nucleophilic sites, capable of attacking electrophilic atoms and acting as hydrogen-bond acceptors [48]. Acetic acid donates hydrogen bonds through its –COOH group to the Cl^-^anion of choline chloride; these hydrogen-bond interactions are responsible for facilitating NADES formation. Furthermore, the free energy of all three conformations was negative, with the third conformation exhibiting the lowest energy, indicating it is the most stable. Additionally, the IGMH scatter plots revealed the presence of weak interactions within the system, including hydrogen bonding and van der Waals forces.

To investigate the effect of ultrasound on the viscosity of NADES, the viscosity of NADES was measured at different temperatures and ultrasound powers. As shown in Table S4, increasing the temperature significantly reduces the viscosity of NADES. Under the condition that thermal effects were excluded using a constant-temperature water bath (Table S5), the viscosity of NADES decreased substantially from 4.8 mPa·s to 2.1 mPa·s as the ultrasonic power increased from 50 W to 100 W, corresponding to a reduction of 56.25%. This indicates that the strong impact and shear forces generated by the collapse of microbubbles in the liquid under ultrasound (i.e., cavitation) lead to a decrease in viscosity [49]. This phenomenon also accounts for the enhanced mass transfer in ultrasound-assisted NADES extraction. According to the Stokes–Einstein equation (D ∝ 1/η), lower viscosity leads to faster molecular motion, allowing the solvent to penetrate more easily into the plant matrix [50]. Meanwhile, reduced viscosity facilitates the formation and collapse of cavitation bubbles, and the strong impact generated upon their collapse promotes faster solvent penetration into the sample, ultimately improving the extraction efficiency of flavonoids.

Furthermore, the morphological features of safflower samples following extraction with various solvents were characterized by SEM, as depicted in Fig. 3a-c**.** The untreated safflower powder exhibited intact cellular structures. Among the solvents tested, however, the NADES-treated group inflicted the most severe damage to the petal cell walls, with obvious cracks and voids clearly visible on the wall surfaces. This induced structural deterioration facilitates solvent ingress and intra-particle mass transfer, thereby boosting the recovery of targeted bioactive constituents. Collectively, NADES, when coupled with ultrasound, exhibits outstanding efficiency in disintegrating safflower cell walls.

**Fig. 3:**
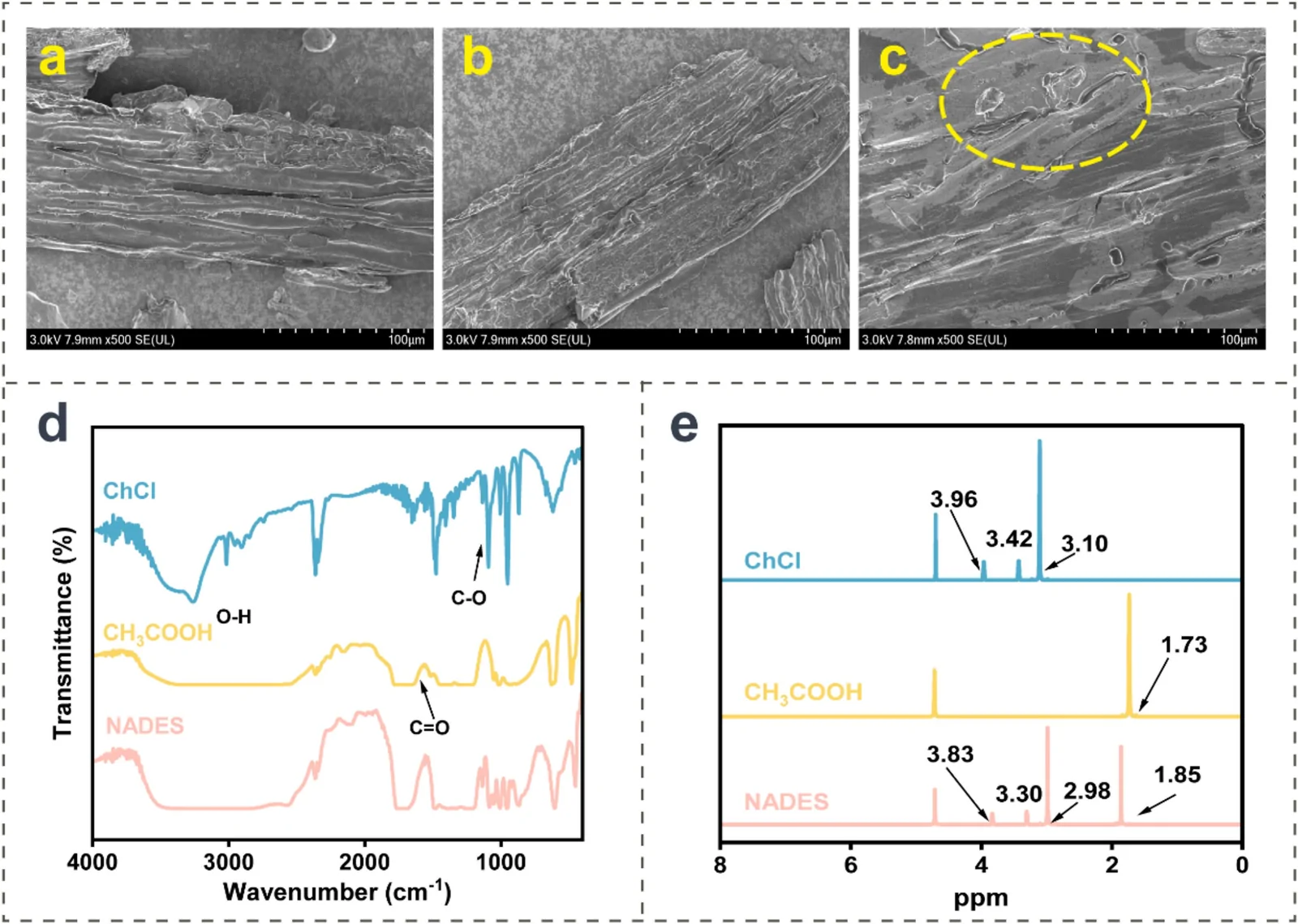
(a) Untreated SEM image of safflower powder; (b) SEM image of safflower powder extracted with 50% ethanol water; (c) SEM image of safflower powder extracted with 50% NADES; (d) FTIR spectra of ChCl, acetic acid, and NADES; (e) ^1^H NMR spectra of ChCl, acetic acid, and NADES.

Fig. 3d shows the FTIR spectrum of NADES. After the formation of a stable liquid NADES system, the O-H stretching vibration peak became broader and more intense, while the C=O stretching vibration peak changed shape and exhibited a red shift. These results indicate the formation of a hydrogen-bond network between choline chloride and acetic acid. According to the ^1^H NMR results (Fig. 3e), the molecular structures of choline chloride and acetic acid remained intact within the NADES system without undergoing chemical reactions, although some characteristic peaks exhibited shifts, further confirming that hydrogen bonds had formed between them.

To further validate the extraction performance of the selected NADES system at the molecular level, MD simulations were employed to determine the optimal conditions for simulating the solvation of quercetin and luteolin. The target molecules were randomly placed in a 10 × 10 × 10 nm cubic simulation box and simulated separately in different solvents: 50% ethanol, 40% NADES, and 50% NADES.

As shown in Fig. 4a and Fig. 4c, the target molecules exhibited different degrees of dispersion during the 0-100 ns simulation period, with the most uniform dispersion observed in the 50% NADES system. Experimental results from real sample analyses also confirmed that this solvent system achieved the best extraction performance. Throughout the 0-100 ns simulation, the RMSD values in the 50% NADES system gradually stabilized and fluctuated within a narrow range, indicating that the target molecules maintained structural stability and that the system reached equilibrium under these conditions. Furthermore, the SASA values under this condition were higher than those in the 50% ethanol–water system, further demonstrating enhanced molecular dispersion and suggesting that the introduction of NADES promoted the dissolution of quercetin and luteolin (Fig. 4b, 4d).

**Fig. 4:**
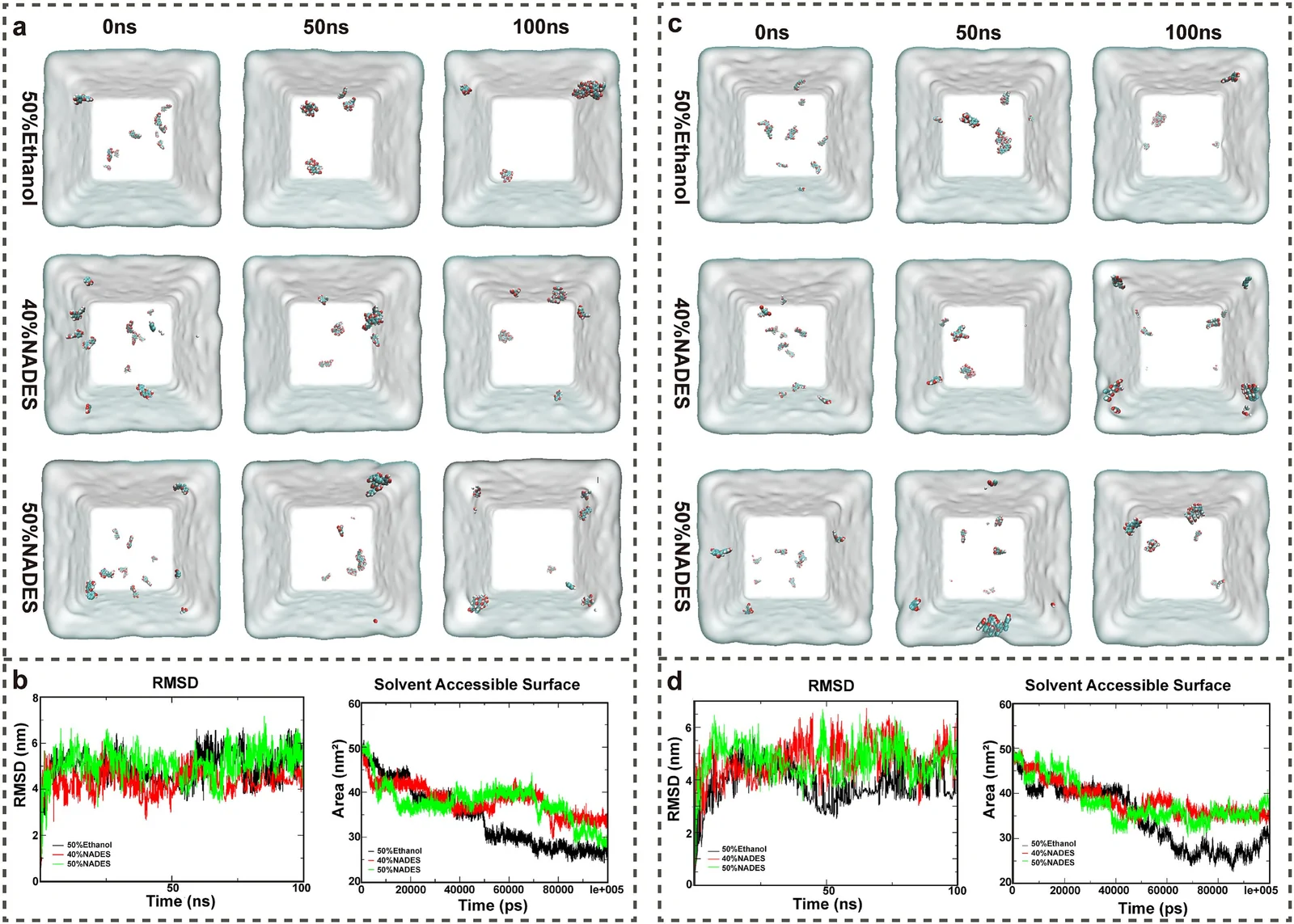
(a) Schematic diagrams of quercetin at 0 ns, 50 ns, and 100 ns under different environments (50% ethanol-water, 40% NADES, and 50% NADES); (b) RMSD and SASA plots of quercetin under different environments; (c) Schematic diagrams of luteolin at 0 ns, 50 ns, and 100 ns under different environments; (d) RMSD and SASA plots of luteolin under different environments.

The results indicate that, among the three conditions tested, quercetin and luteolin achieved an optimal balance between structural stability and efficient solvation in the 50% NADES system. This environment not only effectively prevented molecular structural degradation but also ensured high solvation efficiency, further supporting the experimental findings.

Collectively, these results establish that the choline chloride-acetic acid NADES (50% aqueous solution) serves as an effective green extraction medium for the first step of the separation platform, efficiently releasing target flavonoids from the safflower matrix.

### 3.2 Design and synthesis of the selective adsorbent (SMIP)

To achieve selective recognition of target flavonoids from the complex NADES extract-the second and core module of the separation platform-a surface molecularly imprinted polymer was rationally designed and synthesized. The template-to-monomer ratio was optimized using DFT calculations to maximize the formation of specific recognition sites, and the successful fabrication of SMIP was confirmed through comprehensive material characterization.

During SMIP fabrication, the proportion of template, functional monomer, and crosslinker greatly influences the material’s adsorption behavior. Both insufficient and excessive template-to-monomer ratios reduce specific binding cavities and increase non-specific adsorption, thereby lowering the IF and degrading performance [51]. For this reason, this work employed simulations to determine the optimal ratio of MAA to the template molecule, analyzing their interactions at different molar ratios using IGMH scatter plots. The Gibbs free energy at each molar ratio was calculated to determine the optimal binding ratio between the template molecule and the functional monomer. The relevant data are presented in Table S6. Among the four ratios investigated, the Gibbs free energy reached its lowest value when the molar ratio of the template molecule to MAA was 1:4. To account for solvent effects, SMD calculations were further performed, and the results are shown in Supplementary Table S7 **and** Fig. S2. In solution, only the ΔG for the template-to-monomer ratio of 1:4 remained negative. This behavior can be explained by the balance between cooperative hydrogen bonding and solvation competition. At lower monomer ratios (1:1 and 1:2), the template–monomer complexes are not sufficiently large to form a complete cooperative hydrogen-bonding network, and solvent molecules can compete for the binding sites of quercetin, leading to positive ΔG values. When the ratio reaches 1:4, a sufficiently complete and cooperative hydrogen-bonding network is formed around quercetin, which effectively excludes solvent competition and lowers the free energy to a negative value. Therefore, the 1:4 ratio is thermodynamically the most favorable for preassembly in solution, consistent with the experimentally optimized ratio. Consequently, the ideal molar ratio of the template molecule, functional monomer, and crosslinker was determined to be 1:4:20 in this study.

#### 3.2.1 Molecular mechanism of SMIP recognition

In this work, quantum chemical calculations were employed to simulate the adsorption behavior of the template molecule quercetin on SMIP. The influence of weak interactions, including van der Waals forces, hydrogen bonding, and electrostatic interactions, on the adsorption mechanism of quercetin was systematically investigated, providing deeper insight into the contributions of different functional groups within the adsorption system. ESP is commonly used to identify hydrogen bond donors and acceptors within a system [52]. As shown in Fig. 5a**,** the ESP distribution of MAA and quercetin indicates that the phenolic hydroxyl and carbonyl groups in quercetin exhibit strong electronegativity, enabling quercetin to act as a hydrogen bond acceptor. Additionally, the –COOH functionality of MAA is prone to form hydrogen bonds with the oxygen-bearing groups present in quercetin.

**Fig. 5:**
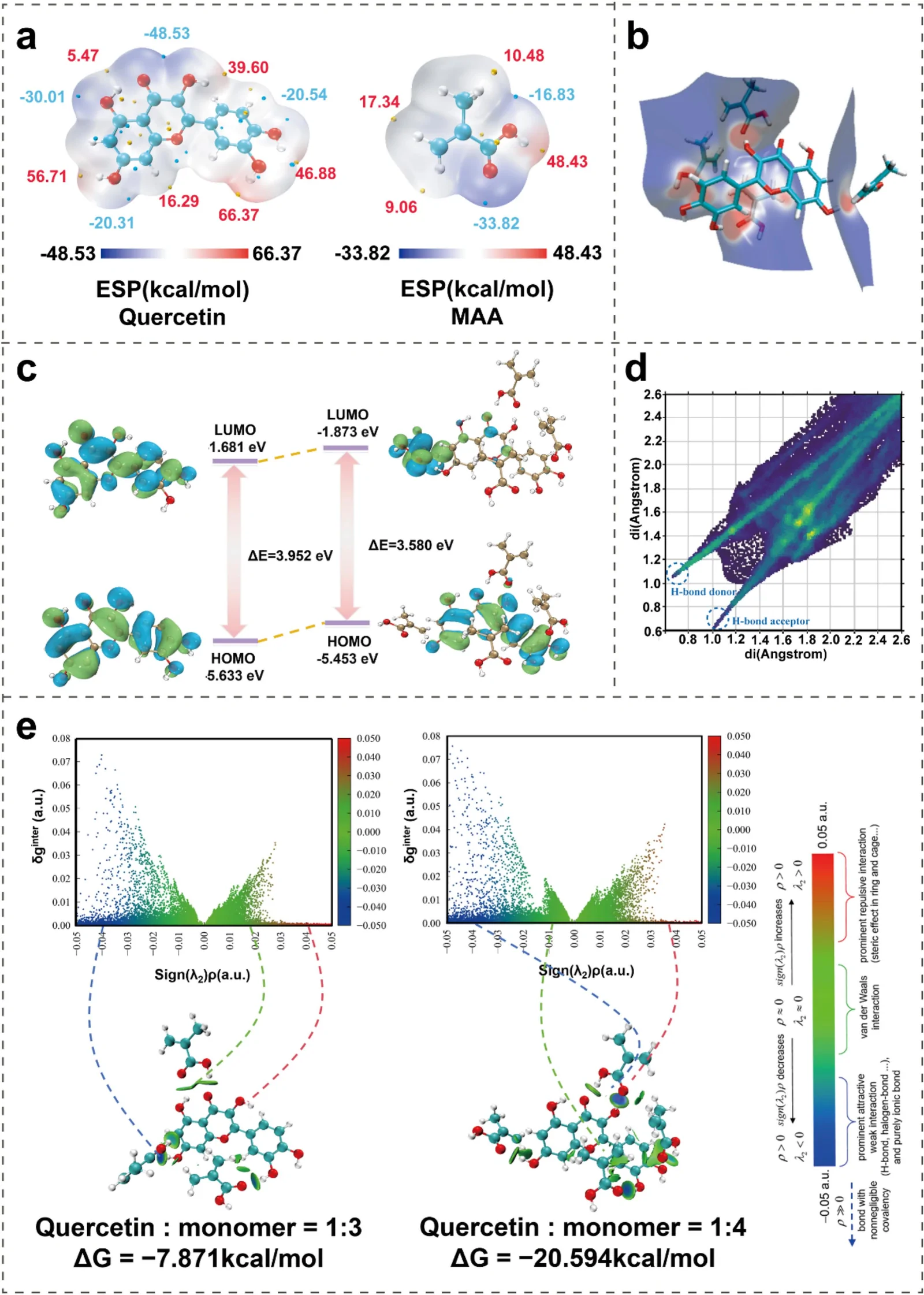
(a) ESP distribution maps of MAA and quercetin; (b) Hirshfeld surface map of quercetin; (c) Energy differences before and after polymer formation; (d) Fingerprint plots of quercetin; (e) IGMH scatter plots and rainbow-colored δg^inter^ isosurfaces of quercetin polymers with different ratios.

Fig. 5b and 5d present the Hirshfeld surface and fingerprint plots of quercetin and MAA, which were employed to clarify the weak interactions between these molecules. These analyses used Dnorm as the mapping function and set the color gradient to transition from blue through white to red [53]. The red regions exhibit smaller Dnorm values, indicating closer intermolecular contacts and stronger interactions. Conversely, van der Waals interactions, which are weaker than hydrogen bonds, are represented by the white regions [54]. Red regions were observed around the oxygen-containing groups of quercetin, indicating higher electron density and a greater tendency to form hydrogen bonds. Meanwhile, the interaction between MAA and quercetin also involved van der Waals forces. This result is consistent with the ESP analysis for adsorption site prediction and the IGMH analysis of hydrogen-bond interactions, both of which indicate that oxygen-containing groups are the primary adsorption sites and can form stronger hydrogen bonds with MAA, thereby enhancing adsorption performance.

Fig. 5e and Fig. S3 present the IGMH maps and isosurface plots of the four polymer systems, revealing various interactions between MAA and quercetin. Hydrogen bonding, van der Waals interactions, and steric hindrance are represented by the blue, green, and red regions in these plots, respectively [55]. Based on the IGMH maps and isosurfaces corresponding to the four different molar ratios, it is clear that hydrogen bonding dominates the adsorption system, with van der Waals interactions playing a secondary role. Specifically, hydrogen–bond interactions are evidenced by a prominent δg^inter^ peak when sign(λ_2_)ρ ranges from -0.05 to -0.03 a.u., while within -0.02 to 0.02 a.u., two small green δg^inter^ peaks reflect van der Waals interactions. In addition, small red δg^inter^ peaks at sign(λ_2_)ρ of 0.02 and 0.03 a.u. indicate steric hindrance effects. These weak interactions work together in the adsorption process; the darker blue isosurfaces and stronger blue scatter–plot peaks further confirm the leading role of hydrogen bonding. The presence of oxygen-containing groups strengthens hydrogen-bond interactions, thereby generating specific recognition sites and more efficient internal recognition driving forces in the polymer.

#### 3.2.2 Material Characterization: Confirming the successful construction of the selective adsorbent

SEM was employed to examine the microscopic structures of SU-102, SMIP, and MIP. Fig. 6b shows images of the five materials, where the incorporation of the SU-102 substrate altered the colors of both MIP and NIP. As shown in Fig. 6c-h, the SEM images of SU-102, SMIP, and MIP clearly demonstrate that SU-102 exhibits a rod-like morphology, whereas SMIP and MIP appear agglomerated. The key difference is that the molecular imprinting layer of SMIP is distributed on the surface of SU-102, while MIP is tightly agglomerated, indicating that the introduction of the substrate alters the distribution of MIP.

**Fig. 6:**
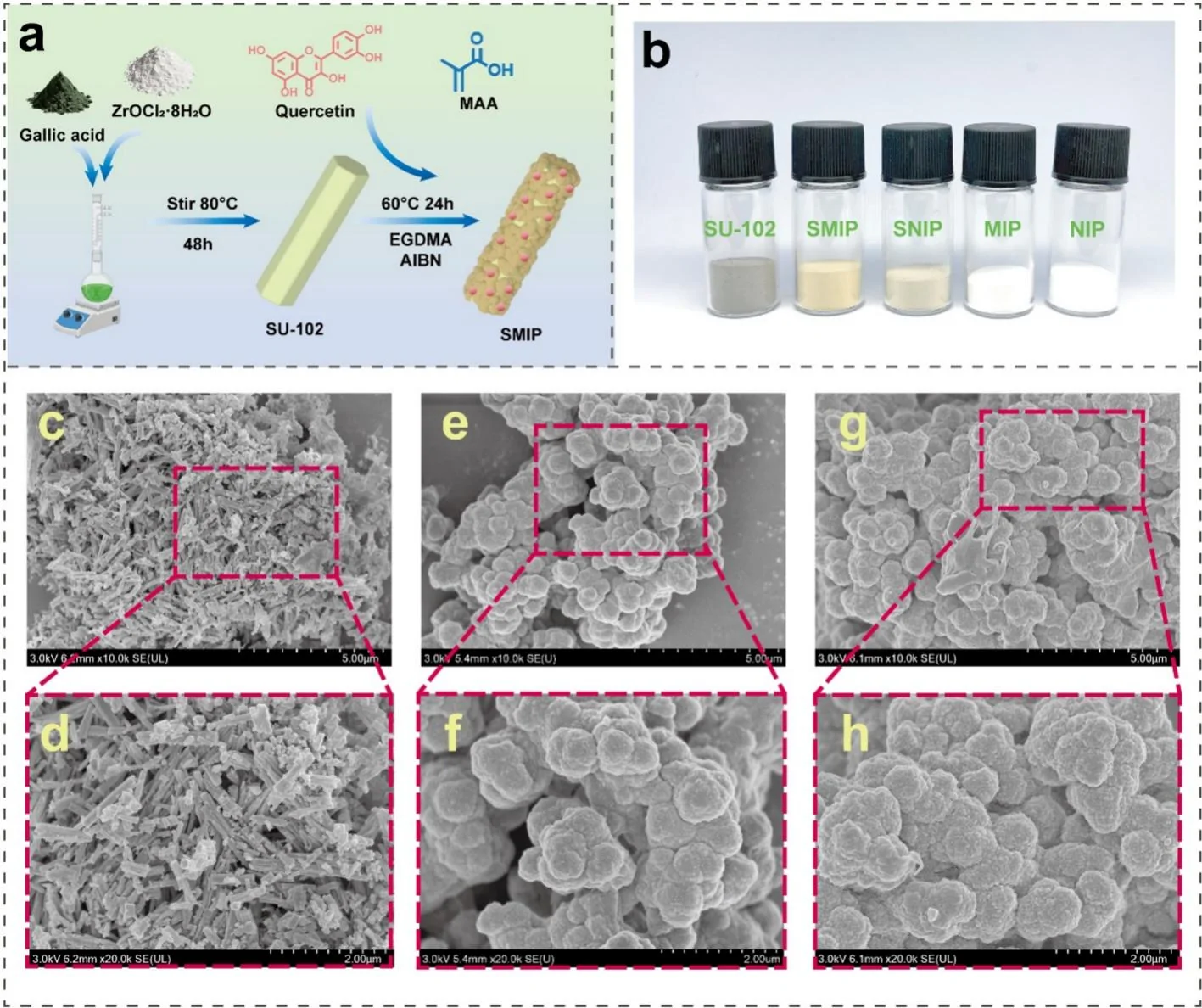
(a) Schematic illustration of SMIP synthesis; (b) Photographs of different materials; (c-d) SEM images of SU-102; (e-f) SEM images of SMIP; (g-h) SEM images of MIP.

To further characterize the pore structures of SU-102, SMIP, and MIP, nitrogen adsorption experiments were conducted to evaluate their porosity and analyze their pore size distributions. BET analysis was performed on SU-102, SMIP, and MIP. The nitrogen adsorption–desorption isotherms of SU-102, SMIP, and MIP are shown in Fig. 7a-c. As illustrated, the adsorption and desorption curves of SMIP and MIP were relatively similar. The structural and property stability of the materials was further evidenced by the fact that, for SU-102, SMIP, and MIP, each desorption curve was higher than its corresponding adsorption curve. Capillary condensation theory accounts for the hysteresis loop observed in the adsorption–desorption isotherms of SMIP and MIP. Nitrogen entry into the pores faced limited resistance, thereby favoring the adsorption. With decreasing pressure, desorption resistance intensified, preventing full desorption. It was only at lower pressures that desorption took place, ultimately producing the hysteresis loop. Conversely, the SU-102 isotherm showed no hysteresis, which may be explained by its carbon-based polymer nature featuring predominantly flexible pores with larger average diameters. In the case of SMIP and MIP, gas adsorption induced pore contraction, which impeded desorption and ultimately produced unclosed isotherms [56], [57], [58]. Pore size analysis of SU-102, SMIP, and MIP showed that their average pore diameters were 14.132 nm, 11.423 nm, and 3.498 nm, respectively (Table S8). Combined with the SEM results, these findings demonstrate that the incorporation of SU-102 improved the pore structure of the MIP.

**Fig. 7:**
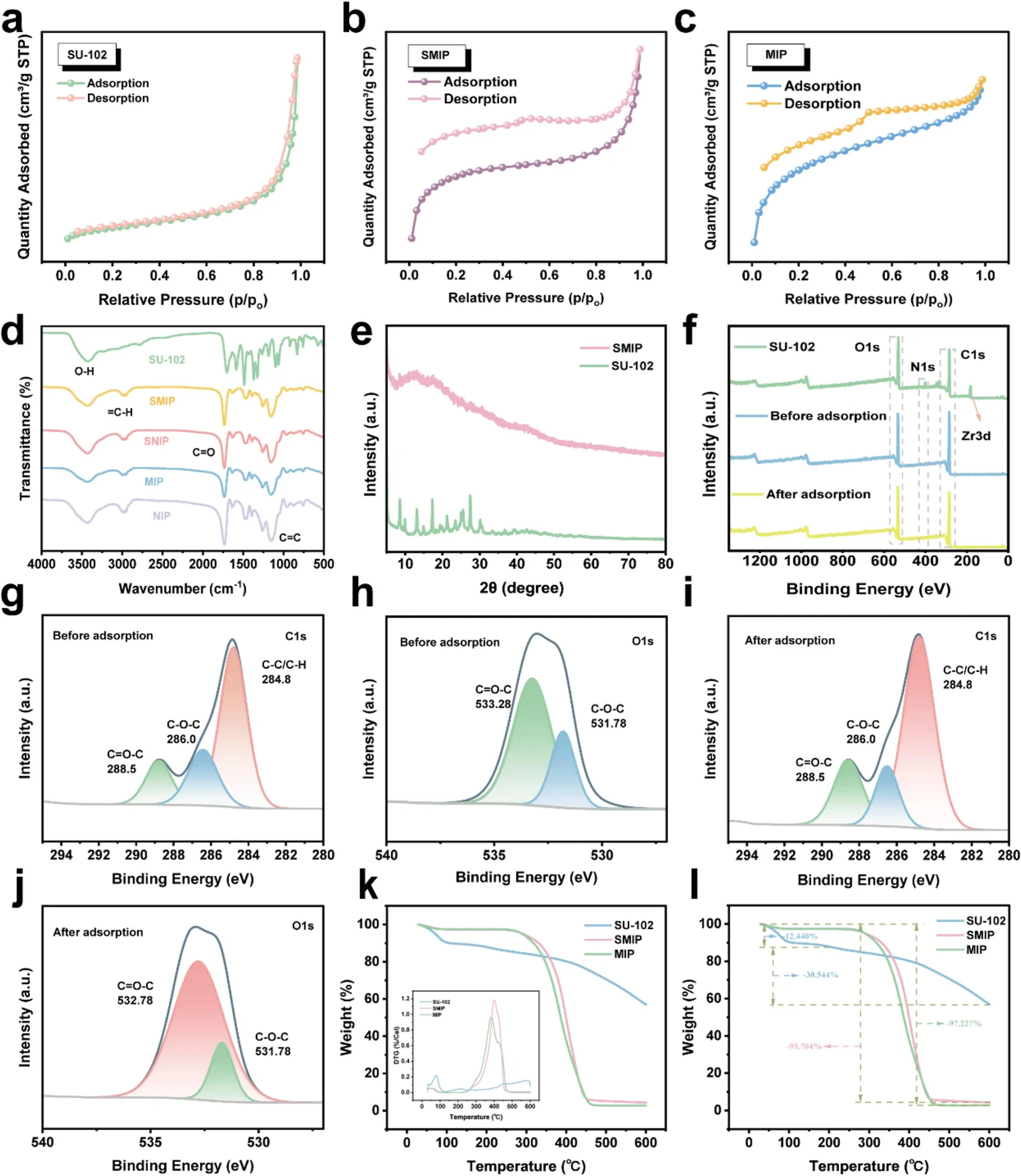
(a-c) Nitrogen adsorption–desorption isotherms of SU-102, SMIP, and MIP; (d) FTIR spectra of SU-102, SMIP, SNIP, MIP, and NIP; (e) XRD patterns of SU-102 and SMIP; (f) XPS survey spectra of SU-102 and SMIP; (g-h) C1s XPS spectra of SMIP before and after adsorption; (i-j) O1s XPS spectra of SMIP before and after adsorption; (k-l) TGA curves of SU-102, SMIP, and MIP (insets show the DTG curves).

To investigate the polymer configurations, FTIR characterization was performed on SU-102, SMIP, SNIP, MIP, and NIP. Around 3400 cm^-^^1^, a peak corresponding to OH bond stretching vibrations appeared, as indicated in Fig. 7d, attributed to the O-H groups in MAA and SU-102. The O-H stretching vibration peak of SU-102 exhibited a stronger signal than those of SMIP and MIP, probably attributable to the establishment of intermolecular hydrogen bonds, which relaxed the bonds and thereby reduced the absorption peak intensity. Meanwhile, a peak attributed to =C-H groups emerged at about 3000 cm^-^^1^, which is associated with the formation of MAA on the supporting material. At 1773 cm^-^^1^, a characteristic C=O absorption band was detected, and the peak intensity of SU-102 was significantly stronger than those of MIP and SMIP, which may be ascribed to the formation of an imprinted layer on the SU-102 surface. These changes further confirm the successful synthesis of the materials. Moreover, the FTIR spectra of SMIP and SNIP exhibited similar peak positions, intensities, and shapes, indicating similar chemical compositions and that the quercetin molecules were successfully removed during preparation. This finding suggests that the template molecules were associated with the functional monomers solely via hydrogen-bond interactions, without participating directly in the polymerization reaction.

As shown in Fig. 7e, the XRD patterns of the SU-102 support and SMIP were analyzed. The main diffraction peaks of SU-102 appeared at 2θ = 8.6°, 13.2°, 17.3°, and 27.5°, along with several minor peaks [58]. In contrast, after imprinting polymerization, the XRD pattern of SMIP exhibited only a broad diffraction peak between 2θ = 15° and 20°, with a significantly reduced peak intensity compared to the support material. The reduced diffraction intensity is likely due to the deposition of a thick imprinted polymer shell on the SU-102 surface during SMIP preparation, which partially masked the crystalline structure and weakened the observed diffraction peaks.

To explore changes in the elemental composition of SU-102 and SMIP, XPS analyses were performed. As shown in Fig. 7f, distinct Zr3d and N1s peaks were clearly observed in the spectrum of SU-102. However, after imprinting polymerization on the support produced SMIP, the relative abundances of both elements dropped significantly. Interestingly, Zr3d and N1s signals were barely observable in SMIP, which can be ascribed to a thick imprinted layer on the SU-102 surface that diminished the detectable content of these elements. This observation provides additional confirmation that SMIP was successfully synthesized and that molecular imprinting was effectively achieved on the SU-102 surface.

TGA analysis was conducted to evaluate the thermal stability of the materials. Measurements were carried out across a temperature range spanning room temperature to 300 °C, and the resulting TGA curves are displayed in Fig. 7k-l. With increasing temperature, the moisture inside the SU-102 particles progressively evaporated, producing multiple weight-loss stages and a total weight loss of 42.984% [58]. In contrast, the SMIP and MIP samples exhibited two stages of weight loss, with total weight-loss percentages of 95.704% and 97.227%, respectively. This significant weight loss was mainly attributed to the evaporation of water, monomers, and solvents trapped within the pores of SMIP and MIP. The similar total weight-loss percentages of SMIP and MIP suggest that an imprinted layer of MIP was successfully formed on the surface of SU-102.

Taken together, the comprehensive characterization results confirm the successful construction of the SMIP selective adsorbent with a well-defined surface-imprinted structure. The incorporation of the SU-102 support not only improved the pore architecture but also provided a platform for distributing recognition sites on the material surface, which is crucial for achieving efficient target capture in the subsequent solid-phase extraction step.

### 3.3 Separation performance of the SMIP-SPE module

To evaluate the effectiveness of SMIP as the selective enrichment component of the separation platform, its adsorption performance was systematically assessed through static adsorption, dynamic adsorption, and selectivity experiments. The influence of key operational parameters-including initial concentration, adsorption time, temperature, pH, and adsorbent dosage-was investigated to establish optimal conditions for the SPE module.

#### 3.3.1 Static Adsorption: Binding capacity and isotherm analysis

The adsorption performance of SMIP was assessed via static adsorption experiments, in which the adsorption capacities of SU-102, SMIP, SNIP, MIP, and NIP were measured under different concentrations of quercetin solutions. Fig. 8b shows that as the concentration increased, the adsorption capacities of all tested materials gradually rose. Over the concentration range of 10-300 μg/mL, SU-102 displayed a rapid increase in adsorption capacity, in contrast to the more gradual increases observed for the other materials. At 350 μg/mL, the adsorption capacities of all materials rose slowly, ultimately reaching saturation at 500 μg/mL. All materials exhibited rapid initial adsorption rates, followed by slower rates as equilibrium was approached. The larger concentration gradient present during the initial stage enhanced the diffusion of quercetin molecules toward the polymer materials, which accounts for this behavior. Adsorption equilibrium was achieved once the concentration exceeded a certain level, as the active sites on the polymer surface became completely saturated with target molecules. Evidently, SU-102 possessed a significantly higher adsorption capacity than the other materials, and SMIP and SNIP showed higher adsorption capacities than MIP and NIP, respectively. These results indicate that the incorporation of SU-102 enhanced the adsorption capacity of conventional molecularly imprinted polymers. Furthermore, differences in adsorption capacities within the same material groups, such as SMIP versus SNIP and MIP versus NIP, further confirm the presence of specific recognition sites. Additionally, the imprinting factor (IF) value of SMIP and SNIP (1.74) was slightly higher than that of MIP and NIP (1.69), which may be attributed to the formation of more effective and higher-affinity recognition sites on the surface of SU-102 through the imprinted layer, rather than relying solely on physical surface area. This result directly validates the advantage of the surface imprinting strategy employed in constructing the separation platform.

**Fig. 8:**
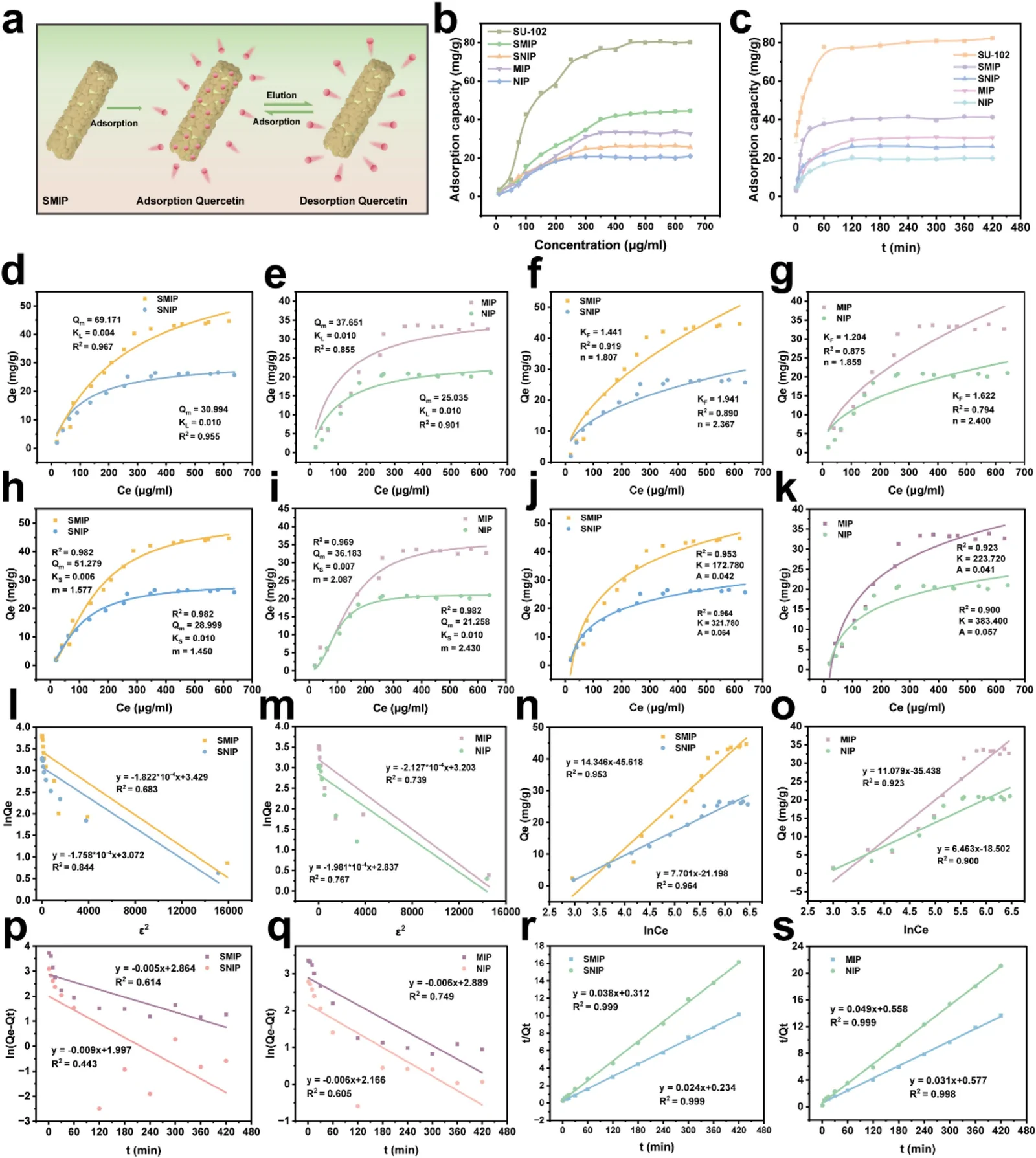
(a) Schematic illustration of the adsorption mechanism of SMIP; (b) Static adsorption curves of SU-102, SMIP, SNIP, MIP, and NIP; (c) Dynamic adsorption curves of the different materials; (d-e) Langmuir fitting plots of SMIP and MIP; (f-g) Freundlich fitting plots of SMIP, SNIP, MIP, and NIP; (h-i) Sips fitting plots of SMIP, SNIP, MIP, and NIP; (j-k) Temkin fitting plots of SMIP, SNIP, MIP, and NIP; (l-m) D-R fitting plots of SMIP, SNIP, MIP, and NIP; (n-o) Linear Temkin fitting plots of SMIP, SNIP, MIP, and NIP; (p-q) Pseudo-first-order kinetic fitting plots of SMIP, SNIP, MIP, and NIP; (r-s) Pseudo-second-order kinetic fitting plots of SMIP, SNIP, MIP, and NIP.

The fitting curves of five adsorption models for SMIP are shown in Fig. 8d-o, with the corresponding parameters listed in Table 3 and 4. For SMIP, the theoretical saturated adsorption capacity (Q_m_) calculated using the Sips model was closest to the actual saturated adsorption capacity of the adsorbent toward the target molecule (44.639 mg/g). In addition, the correlation coefficient (R^2^) values obtained from fitting the different models were as follows: Sips model (R^2^ = 0.982), Langmuir (R^2^ = 0.967), Temkin (R^2^ = 0.953), Freundlich (R^2^ = 0.919), and the D-R model, which showed the poorest fit (R^2^ = 0.683). The Sips model demonstrated the strongest correlation among the tested models. Similarly, for SNIP, MIP, and NIP, the R^2^ values obtained from the Sips model fitting exceeded those from the other adsorption models. Therefore, the Sips model more effectively describes the adsorption of target molecules by SMIP. The fitting results of the Sips model showed that the heterogeneity parameter m = 1.577 > 1, indicating that the adsorption mechanism of SMIP toward the target molecules is dominated by monolayer adsorption occurring mainly on a heterogeneous surface, with a finite saturation capacity [59], [60]. Multiple interactions, such as hydrogen bonding, van der Waals forces, and electrostatic interactions, may be involved in the adsorption process of SMIP, a finding that agrees with the DFT-predicted adsorption mechanism.

**Table 3:** Adsorption isotherm constants from Langmuir and Freundlich fitting.

Materials	Langmuir model			Freundlich model		
	Q_m_ (mg/g)	K_L_(L/mg)	R_1_^2^	K_F_	n	R_2_^2^
SMIP	69.171	0.004	0.967	1.441	1.807	0.919
SNIP	30.994	0.010	0.955	1.941	2.367	0.890
MIP	37.651	0.010	0.855	1.204	1.859	0.875
NIP	25.035	0.010	0.901	1.622	2.400	0.794

**Table 4:** Adsorption isotherm constants for different isotherm models. **Table 5.** Kinetic fitting parameters of SMIP.

Materials	Isotherm	Parameter	Value	Parameter	Value	Parameter	Value	R^2^
SMIP	Sips	K_s_	0.006	Q_m_	51.279	m	1.577	0.982
	D-R	K	1.822*10^-4^	Q_m_	30.850	−	−	0.683
	Temkin	K	172.780	A	0.042	−	−	0.953
SNIP	Sips	K_s_	0.010	Q_m_	28.999	m	1.450	0.982
	D-R	K	1.758*10^-4^	Q_m_	21.589	−	−	0.844
	Temkin	K	321.780	A	0.064	−	−	0.964
MIP	Sips	K_s_	0.007	Q_m_	36.183	m	2.087	0.969
	D-R	K	2.127*10^-4^	Q_m_	24.607	−	−	0.739
	Temkin	K	223.720	A	0.041	−	−	0.923
NIP	Sips	K_s_	0.010	Q_m_	21.258	m	2.430	0.982
	D-R	K	1.981*10^-4^	Q_m_	17.065	−	−	0.767
	Temkin	K	383.400	A	0.057	−	−	0.900

#### 3.3.2 Dynamic Adsorption: Kinetics and mass transfer

Dynamic adsorption experiments were carried out to further evaluate the adsorption performance of SMIP by studying the effect of adsorption time on the adsorption capacities of SU-102, SMIP, SNIP, MIP, and NIP. The adsorption capacity of each material increased slowly over time, attaining equilibrium at roughly 180 min, as seen in Fig. 8c. The adsorption capacities followed the order: Q_SU-102_ > Q_SMIP_ > Q_MIP_ > Q_SNIP_ > Q_NIP_. Notably, the adsorption capacities increased most rapidly within the first 0–30 min, with SMIP and MIP exhibiting markedly greater adsorption efficiencies than SNIP and NIP. This behavior can be attributed to the abundance of unoccupied imprinted sites in SMIP and MIP. As adsorption time progressed, the number of available imprinted sites gradually decreased until equilibrium was achieved.

To further investigate the dynamic adsorption mechanism of SMIP, the adsorption data for the five materials were fitted using kinetic models. The fitting curves are shown in Fig. 8p-s, and the corresponding parameters for the pseudo-first-order and pseudo-second-order kinetic models are listed in Table 5. With an R^2^ value of 0.999, the pseudo-second-order model for SMIP demonstrated a notably strong correlation. Moreover, the theoretical saturated adsorption capacity (Q_e_) calculated using the pseudo-second-order model closely matched the actual saturated adsorption capacity. According to these results, a rate-limiting step involving either chemical reactions or chemisorption, along with electron sharing and exchange, governs the adsorption of target molecules onto SMIP [61]. A higher Q_e_ value was obtained for SMIP than for SNIP using the pseudo-second-order model, which points to SMIP's excellent specific adsorption capacity.

**Table 5:** Kinetic fitting parameters of SMIP.

Materials	Pseudo-first-order kinetic model			Pseudo-second-order kinetic model		
	Q_e,cal_ (mg/g)	K_1_	R_1_^2^	Q_e,cal_ (mg/g)	K_2_	R_2_^2^
SMIP	17.53	0.005	0.614	42.409	0.024	0.999
SNIP	7.364	0.009	0.443	26.638	0.038	0.999
MIP	17.981	0.006	0.749	32.478	0.031	0.998
NIP	8.720	0.006	0.605	20.513	0.048	0.999

#### 3.3.3 Selectivity: Specific recognition of flavonoid aglycones

To investigate the specific adsorption capability of SMIP toward flavonoids, selectivity studies were performed with structurally similar compounds, and interfering components present in real samples of safflower. These included ferulic acid, syringic acid, and protocatechuic acid, as well as structural analogs such as kaempferol, apigenin, and luteolin. Under identical adsorption conditions, the adsorption capacities of SMIP and SNIP toward these different compounds were shown in Fig. 9a. It was observed that SMIP exhibited significantly higher adsorption capacities for flavonoids compared to the other compounds, with the highest imprinting factor reaching 1.80. Ferulic acid, syringic acid, and protocatechuic acid, which are other constituents of safflower, were used as adsorption interferents in this study. Due to their substantial structural differences from quercetin, their adsorption capacities were lower than that of quercetin. Kaempferol, apigenin, and luteolin exhibited higher adsorption capacities than the three non-flavonoid compounds mentioned above because they possess structures and functional groups similar to those of quercetin. The IF values of SMIP toward the different compounds were calculated according to the imprinting factor equation, and the results are listed in Table 6 as follows: IF_Kaempferol_ (1.80) > IF_Quercetin_ (1.74) > IF_Apigenin_ (1.71) > IF_Luteolin_ (1.59) > IF_Ferulic Acid_ (1.18) > IF_Protocatechuic Acid_ (1.01) > IF_Syringic Acid_ (0.72). The imprinting factor reflects the degree of adsorption enhancement of the imprinted material relative to the non-imprinted material, rather than the absolute adsorption capacity. Although the quercetin-templated SMIP exhibited a relatively high absolute adsorption capacity for quercetin, quercetin contains 3,5,7,3′,4′-pentahydroxy groups, among which the *ortho*-dihydroxy group on the B ring (3′,4′-OH) provides stronger non-specific hydrogen-bonding interactions. This also leads to relatively high adsorption of quercetin on SNIP, thereby reducing its IF. In comparison, kaempferol lacks the 3′-OH on the B ring, resulting in lower steric hindrance and easier entry of the molecule into the cavities formed by quercetin imprinting [62]; meanwhile, its lower number of phenolic hydroxyl groups leads to weaker non-specific adsorption, thus giving a higher IF. This indicates that the higher IF of kaempferol mainly originates from its lower steric hindrance and weaker non-specific adsorption, rather than from a stronger specific affinity for the imprinted cavities than quercetin. Therefore, the selective recognition ability of SMIP should be comprehensively evaluated by combining absolute adsorption capacity and selectivity coefficient. These results demonstrate that SMIP exhibits excellent selectivity toward flavonoid compounds. The high selectivity arises from the imprinting cavities created during synthesis, which are complementary in size, shape, and functional group orientation to the template molecule. This selectivity is the key feature that enables the SMIP-SPE module to effectively isolate target flavonoids from the complex NADES extract, addressing the inherent selectivity limitation of NADES extraction alone.

**Fig. 9:**
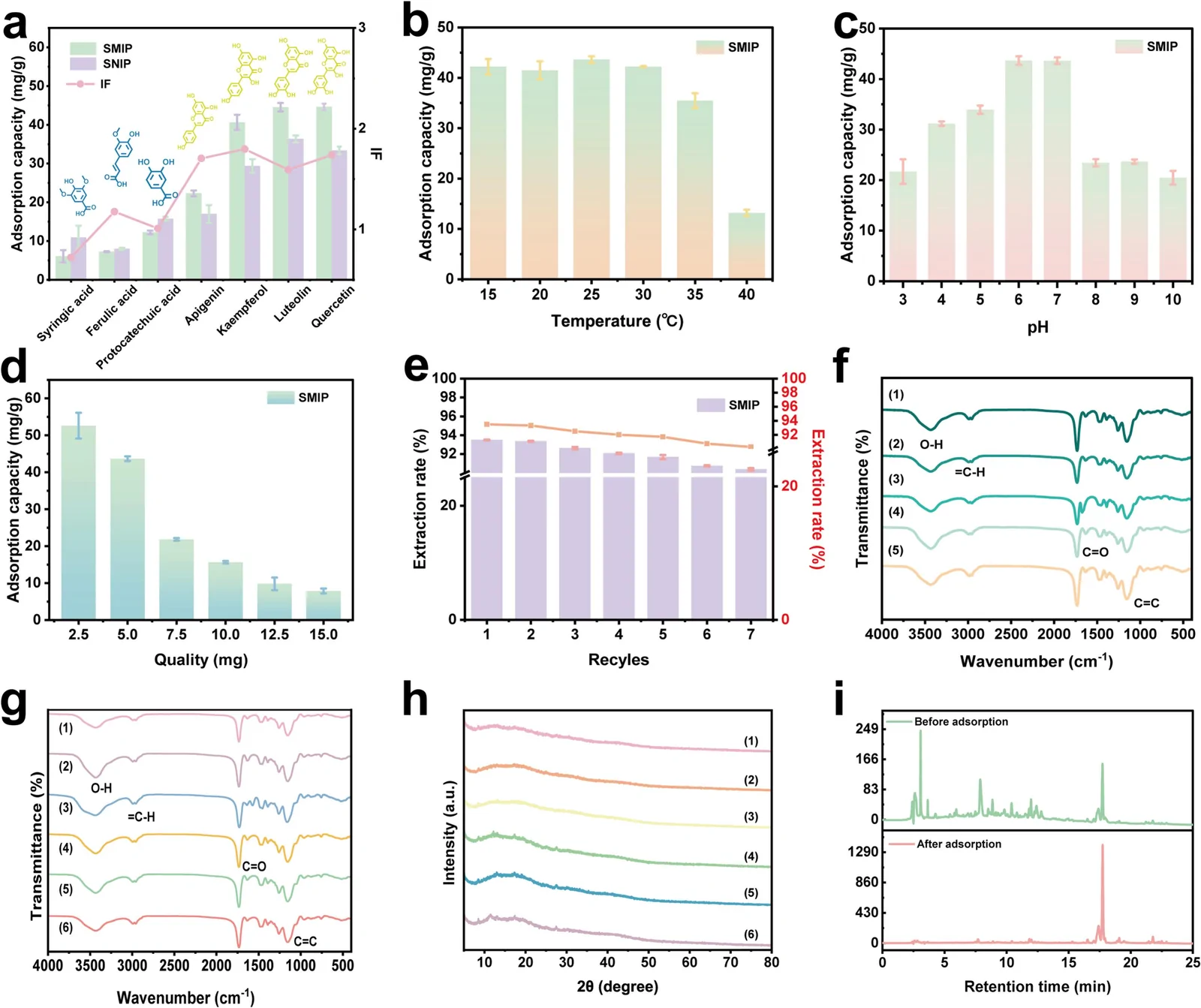
(a) Selectivity results of SMIP and SNIP; (b) Effects of temperature on the adsorption performance of SMIP; (c) Effects of pH on the adsorption capacity of SMIP; (d) Effects of SMIP dosage on adsorption capacity; (e) Reusability of SMIP; (f) FTIR spectra of SMIP after the 1st, 2nd, 4th, 5th, and 7th cycles of use; (g-h) XRD and FTIR spectra of (1) SMIP, SMIP after immersion for 24 h in (2) HCl solution at pH = 1, (3) NaOH solution at pH = 13, (4) methanol, (5) ethyl acetate, and (6) dichloromethane; (i) Comparative HPLC chromatograms of safflower extract before and after adsorption by SMIP.

**Table 6:** Adsorption data of each compound.

Molecules	Q_SMIP_ (mg/g)	Q_SNIP_ (mg/g)	IF	SC
Syringic acid	6.065	8.410	0.72	0.41
Ferulic Acid	7.252	6.161	1.18	0.68
Protocatechuic Acid	12.216	12.123	1.01	0.58
Apigenin	22.315	13.084	1.71	0.98
Kaempferol	40.651	22.611	1.80	1.03
Luteolin	44.546	27.962	1.59	0.92
Quercetin	44.639	25.683	1.74	

As shown in Fig. S4 **and** Table S9, in the mixed standard solution, the adsorption capacity of SMIP toward the target compounds showed significant differences. The adsorption amounts of flavonoids (luteolin, quercetin, apigenin, and kaempferol) were much higher than those of phenolic acids. During competitive adsorption, SMIP exhibited stronger affinity for flavonoids. This is mainly attributed to the successful formation of imprinted cavities in SMIP.

#### 3.3.4 Optimization of operational parameters

Temperature is a crucial factor influencing the adsorption performance of SMIP; therefore, the effects of different temperatures were investigated. As shown in Fig. 9b, the material exhibited optimal adsorption performance at room temperature (25 °C). As the temperature increased from 15 °C to 25 °C, no significant difference in adsorption capacity was observed. However, when the temperature reached 35 °C, the adsorption capacity decreased. This may be attributed to the enhanced thermal motion of molecules during the adsorption process, which weakened the intermolecular interactions (e.g., hydrogen bonding) between quercetin and SMIP, thereby reducing the adsorption capacity [63]. Consequently, 25 °C was selected for subsequent experiments.

pH can influence both the stability of quercetin molecules and the adsorption performance of SMIP toward quercetin. Therefore, to obtain the optimal adsorption conditions, this study investigated the adsorption capacities of SMIP for quercetin under various pH levels. Fig. 9c illustrates the changes in SMIP’s adsorption capacity toward quercetin across different pH values. As the pH of the quercetin solution increased from 3 to 5, the adsorption capacity gradually improved. When the environment is strongly acidic, protons outcompete the hydrogen atoms that form hydrogen bonds within the material. This competition causes those hydrogen atoms to become protonated, thereby reducing the hydrogen-bond interactions between quercetin and the material, which reduces the adsorption capacities of both SMIP and SNIP. When the pH increased to 6 or 7, the materials’ adsorption capacities reached their maximum. However, at pH 8, the adsorption capacity began to decline, dropping sharply at pH 9 ∼ 10. This decline occurs because quercetin is fully dissociated under alkaline conditions, existing as an anion with enhanced water solubility, while the surfaces of SMIP and SNIP contain abundant carboxyl groups. When the pH continues to rise, OH^–^ ions in the solution easily outcompete the hydrogen atoms that participate in hydrogen bonds, thereby breaking the existing hydrogen bonds and resulting in a lower adsorption capacity [64]. Considering that pH adjustment can be cumbersome in practical applications, a pH of 7 was selected for subsequent experiments.

This experiment investigated the effect of the adsorbent mass of SMIP on its adsorption capacity. Fig. 9d reveals that increasing the SMIP mass led to a gradual reduction in adsorption capacity. This is attributed to adsorption being primarily driven by concentration differences: when the adsorbent amount is small, the adsorption sites on each milligram of SMIP are fully utilized; as the adsorbent amount increases, the number of adsorption sites becomes excessive, leading to a decrease in adsorption capacity. Considering that weighing 2.5 mg may result in larger experimental errors, a SMIP mass of 5 mg was ultimately selected.

### 3.4 Machine Learning-Assisted optimization of the separation process

To examine the relative contribution of each operational parameter to the separation performance, machine learning was employed as an auxiliary exploratory tool. This data-driven approach complements the experimental optimization in Section 3.3.4 by providing interpretable insights into the adsorption process.

In this study, ten machine learning models were developed to predict the adsorption capacity of SMIP toward quercetin, and their performances were comprehensively evaluated based on the R^2^ values of the training and testing sets. Fig. 10a presents a comparison of R^2^ values among the different models. The results showed that the RF model achieved relatively high R^2^ values on both the training and testing sets, with only a small difference between them, indicating good fitting performance. Its predictive capability was also superior to that of the other models, primarily because RF constructs multiple decorrelated decision trees through bootstrap aggregation and feature randomness and then generates the final prediction by averaging the outputs (Fig. S5 and S6). This mechanism significantly reduces the overfitting of individual decision trees to local fluctuations and noise in the training data, enabling the model to capture the underlying patterns in adsorption experiments while remaining robust to noisy data, thereby achieving excellent generalization performance [65]. SHAP analysis and the feature correlation heatmap quantified the influence of each adsorption condition, enabling accurate prediction and providing interpretable evidence for experimental optimization. Therefore, the RF model was selected for subsequent studies.

**Fig. 10:**
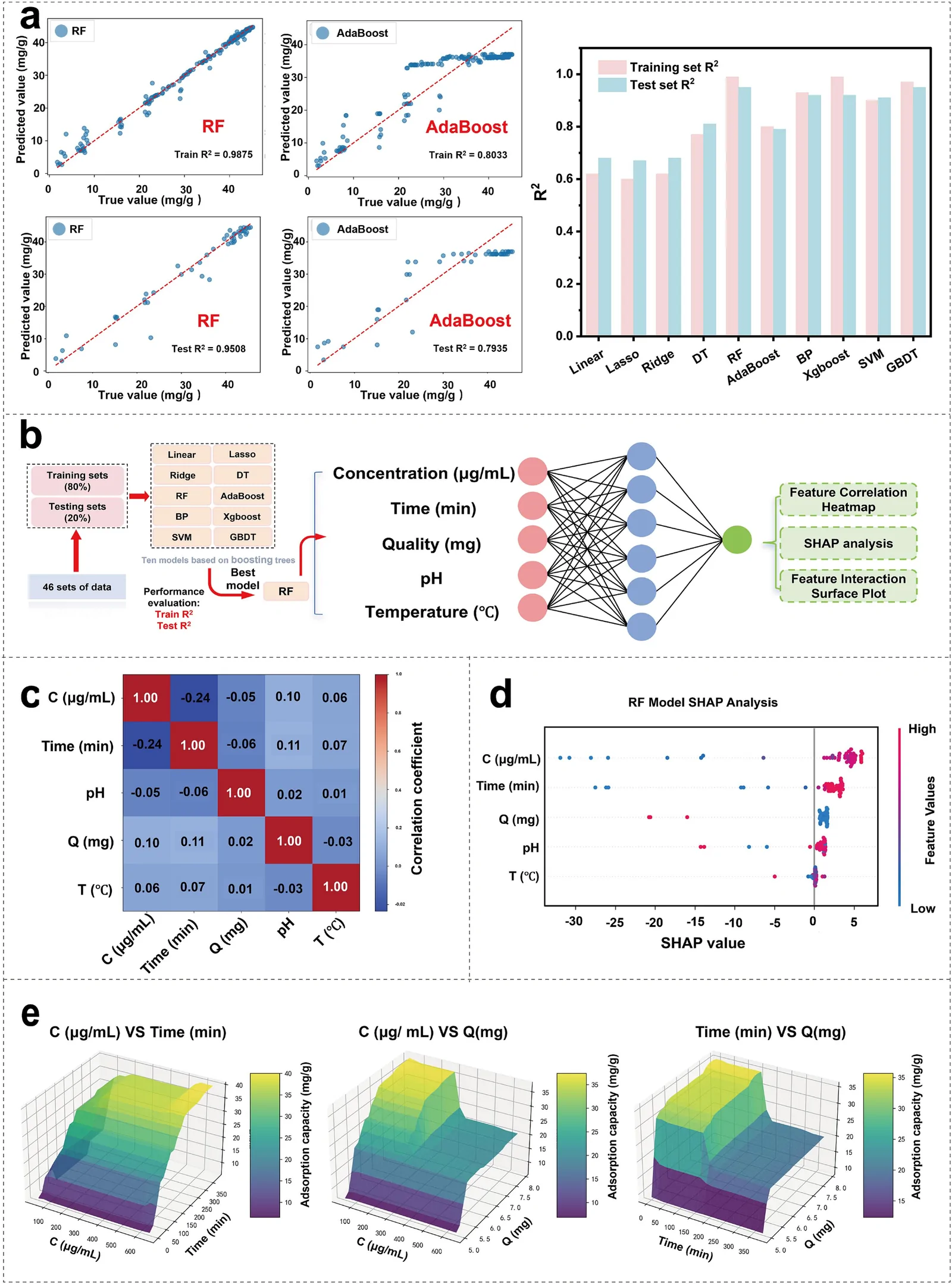
(a) Partial model residual plots and comparison of R^2^ values among different models; (b) Flowchart of the machine learning workflow; (c) Feature correlation heatmap (Red and blue represent positive and negative correlations, respectively); (d) SHAP value analysis (Positive and negative SHAP values indicate positive and negative contributions of each influencing factor to the prediction result, respectively); (e) Feature interaction surface plots(Interactive effects of two influencing factors on the adsorption capacity).

To further elucidate the effects of various adsorption conditions on the adsorption capacity of quercetin, feature correlation heatmaps, SHAP value analyses, and feature interaction surface plots based on the RF model were employed to analyze the relationships between adsorption conditions (initial concentration, temperature, time, pH, and adsorbent dosage) and adsorption capacity (Fig. 10c-e). The SHAP analysis (Fig. 10d) quantitatively evaluated the influence of these conditions on quercetin’s adsorption capacity. As shown in Fig. 10d, the initial concentration of the target molecule was identified as the most influential factor, followed by adsorption time. Except for the adsorbent dosage, the remaining factors exhibited positive SHAP values, indicating positive correlations with the adsorption capacity. These findings provide a prioritized basis for the subsequent optimization of adsorption conditions. The feature correlation heatmap illustrated the linear correlations among the adsorption conditions. As shown in Fig. 10c, the absolute correlation coefficients among all conditions were below 0.24. Only the initial concentration and adsorption time exhibited a weak correlation (absolute value = 0.24), whereas the absolute correlation coefficients among the remaining features were all ≤ 0.11. These results indicate that the features were largely independent of one another and could therefore be used as independent variables in subsequent modeling. Fig. 10e illustrates the interactive effects of three experimental conditions on the quercetin’s adsorption capacity in this study. When the initial concentration ranged from 500 to 650 μg·mL^-^^1^ and the adsorption time was between 350 and 400 min, a yellow high-value region appeared, corresponding to a significant increase in adsorption capacity. Overall, the different color regions clearly delineated the conditional boundaries between low adsorption capacity (purple region) and high adsorption capacity (yellow/green region). These analyses not only clarified the individual effects of each factor but also revealed the regulatory patterns arising from combinations of conditions, thereby providing a visual reference for optimizing the adsorption process of quercetin onto SMIP. The ML results confirmed that the experimental conditions identified through conventional optimization (Section 3.3.4) are indeed near-optimal, while also revealing the relative importance of each parameter for future method development.

Overall, the machine learning analysis serves only as an auxiliary exploratory tool for summarizing the contributions of variables. The adsorption behavior of SMIP is mainly interpreted based on adsorption isotherms, kinetic experiments, pH-dependent adsorption, temperature-effect experiments, adsorbent mass-effect experiments, selectivity evaluation, and practical sample application results.

### 3.5 Practical Applicability: Reusability, Stability, and real sample validation

To assess the practical viability of the integrated NADES-SMIP-SPE separation platform, the reusability and chemical stability of the SMIP adsorbent were evaluated. Most importantly, the platform was validated by applying it to real safflower samples, demonstrating its ability to selectively enrich target flavonoids from a complex plant matrix.

#### 3.5.1 Reusability and stability of the SMIP adsorbent

The reusability of a material is a crucial factor in evaluating its practical application potential. In this study, the repeated adsorption performance of SMIP toward quercetin was assessed using a solid-phase extraction setup. As shown in Fig. 9e and Table S10, after seven adsorption–desorption cycles, the recovery efficiency of SMIP toward quercetin decreased from 93.50 % to 90.38 %. From cycle one to cycle seven, the removal efficiency decreased by merely 3.12 %, with the final efficiency still exceeding 90 %. SMIP retained high quercetin adsorption performance over seven adsorption–desorption cycles, reflecting its favorable reusability and mechanical stability. In comparison, the original XRD patterns and FTIR spectra of SMIP showed no significant changes after immersion in different solvents (Fig. 9f-h). To directly verify whether the crystalline core of SU-102 was retained in SMIP, we supplemented TEM images of SU-102, non-acid-washed SMIP, and acid-washed SMIP. As shown in Fig. S7, SU-102 exhibited a rod-like morphology. For both the non-acid-washed and acid-washed SMIP, an amorphous polymer shell was observed on the outer layer, while the images showed that the rod-like morphology of SU-102 was retained, further demonstrating that the crystalline framework of SU-102 was preserved in SMIP. These results directly indicate that an intact crystalline SU-102 core remains beneath the polymer shell. Strong acids, strong bases, and related organic solvents barely affected the structural stability of SMIP, providing additional evidence of its superior stability.

#### 3.5.2 Real sample Application: Validation of the integrated separation platform

After analyzing the safflower extract using UPLC-Q-TOF, ten major components were identified. Detailed information is listed in Table 7, including flavonoid compounds (hydroxysafflor yellow A, rutin, luteolin, and quercetin), organic acids (citric acid, protocatechuic acid, and ferulic acid) and amino acid (valine). This compositional analysis confirms the complexity of the safflower matrix and underscores the need for a selective enrichment step.

**Table 7:** Results of UPLC-Q-TOF-MS compositional analysis.

Serial number	Compound name	Ion mode	Retention time	Molecular formula	*m*/*z*	Quality error ppm	Secondary fragment ion
1	Citric acid	−	1.512	C_6_H_8_O_7_	175.1187	1.18	175.1196
2	Protocatechuic acid	−	4.182	C_7_H_6_O_4_	153.0188	3.46	109.0291, 153.0186
3	Valine	+	5.67	C_5_H_11_NO_2_	118.0861	1.32	120.0443, 79.9420
4	Hydroxysafflor yellow A	−	7.95	C_27_H_31_O_17_	611.1597	3.36	491.1198, 287.0574
5	Rutin hydrate	−	9.177	C_27_H_30_O_16_	609.1446	2.47	521.9076
6	Quercetin	+	9.477	C_15_H_10_O_7_	303.0495	1.42	134.5487
7	6-Hydroxykaempferol 3-O-β-D-glucoside	−	9.513	C_21_H_20_O_12_	463.0869	2.15	467.3227
8	Ferulic acid	+	9.665	C_10_H_10_O_4_	195.0662	-5.23	160.8416, 195.0658
9	Luteolin	−	11.173	C_15_H_10_O_6_	285.0398	2.31	135.0105
10	Kaempferol-3-O-rutinoside	−	11.457	C_27_H_30_O_15_	593.1498	2.35	597.1629

To evaluate the specific selectivity of the prepared SMIP toward target compounds in real samples, the chromatograms of safflower extracts before and after treatment with the SPE column were compared (Fig. 9i**)**. The results showed that before SPE treatment, the extract contained numerous impurities, the chromatographic baseline was elevated, and the target peaks were relatively low. After adsorption with the SPE column, the impurity peaks were significantly reduced, the chromatographic baseline stabilized, the target peaks became clearly discernible, and the target compounds were effectively enriched. These findings clearly demonstrate that the prepared SMIP possesses excellent selective recognition ability toward luteolin and quercetin in safflower, enabling effective capture and enrichment of the target compounds from complex matrices while removing most interfering components, thereby significantly improving detection accuracy and sensitivity.

Based on the results above, this study established an extraction method using choline chloride-acetic acid combined with molecularly imprinted solid-phase extraction technology to analyze real safflower samples. Under optimized conditions-specifically, a NADES composed of 50% choline chloride-acetic acid-the extraction efficiency of the target compounds was maximized, as further validated by MD simulations. Following selective adsorption by SPE, the interference from complex matrices was effectively eliminated, and the target compounds were efficiently enriched. This method was successfully applied to safflower samples, demonstrating its feasibility and reliability for detecting target compounds in complex real samples.

### 3.6 Integrated separation Mechanism: A multi-scale Perspective

The collective results from the preceding sections can be synthesized into a coherent mechanistic picture of the NADES-SMIP-SPE separation platform, wherein hydrogen bonding emerges as the central driving force governing the entire separation workflow across three interconnected scales.

At the molecular scale, DFT calculations and MD simulations consistently confirm that hydrogen bonding is the predominant intermolecular interaction governing both NADES extraction and SMIP recognition. The oxygen‑containing groups of flavonoid aglycones form complementary donor–acceptor pairings with the carboxyl/hydroxyl functionalities of NADES components and with MAA, providing a molecular‑level explanation for the strong binding affinities observed experimentally. This mechanistic insight elucidates why the choline chloride-acetic acid NADES exhibits superior extraction performance and why the quercetin‑imprinted SMIP displays high selectivity toward flavonoid targets.

At the material scale, the introduction of SU‑102 support fundamentally improves the separation performance. The surface‑imprinted architecture offers readily accessible recognition sites and enhanced mass‑transfer properties, as reflected in its improved pore structure relative to conventional MIP. This structural advantage directly translates into higher adsorption capacity and a higher imprinting factor, thereby validating the rationale of employing a MOF as the support for surface imprinting.

At the process scale, the integration of NADES extraction with SMIP‑SPE compensates for the limitations of each individual technique. NADES enables the green and efficient extraction of total flavonoids from safflower but inherently lacks selectivity; the SMIP‑SPE module, in turn, confers the necessary specificity to capture the target flavonoids and eliminate interference from co‑extracted matrix components. Machine‑learning analysis further optimises the operating parameters and quantitatively identifies the most critical factors affecting adsorption performance.

Multiscale analysis consistently indicates that hydrogen bonding serves as the key driving force across all levels: it governs NADES formation and flavonoid dissolution, directs template-monomer assembly during SMIP synthesis, enables selective rebinding in the SPE process, and is modulated by operational conditions such as pH and temperature. This mechanistic understanding not only corroborates the experimental observations but also provides a rational theoretical foundation for extending the present platform to the selective recovery of other high‑value phytochemicals from complex medicinal plant matrices.

### 3.7 Greenness evaluation of the method

The greenness of the NADES-SMIP-SPE method was assessed via AGREE and Complex GAPI as per standard protocols. The AGREE score of 0.68 surpassed the 0.6 benchmark, denoting excellent greenness (Fig. 11a), while Complex GAPI showed only three red indicators (Fig. 11b) [66]. In the lower segment of the hexagon on the right side of the original GAPI (waste treatment): NADES is not recovered after separation of the extract; In the area below the central pentagon and above the additional hexagon (yield): the yield of flavonoid aglycones is less than 70%; In the upper left of the additional hexagon (purity): the extract contains organic acids, flavonoids, and other compounds, with a purity of less than 97% [67]. The method’s low consumption of samples and solvents, simplified procedure, and a natural HBA/HBD/water extraction medium led to minimal waste (E-factor = 0.04). Overall, it effectively curtails organic solvent usage and carries negligible risks to humans and the environment [68].

**Fig. 11:**
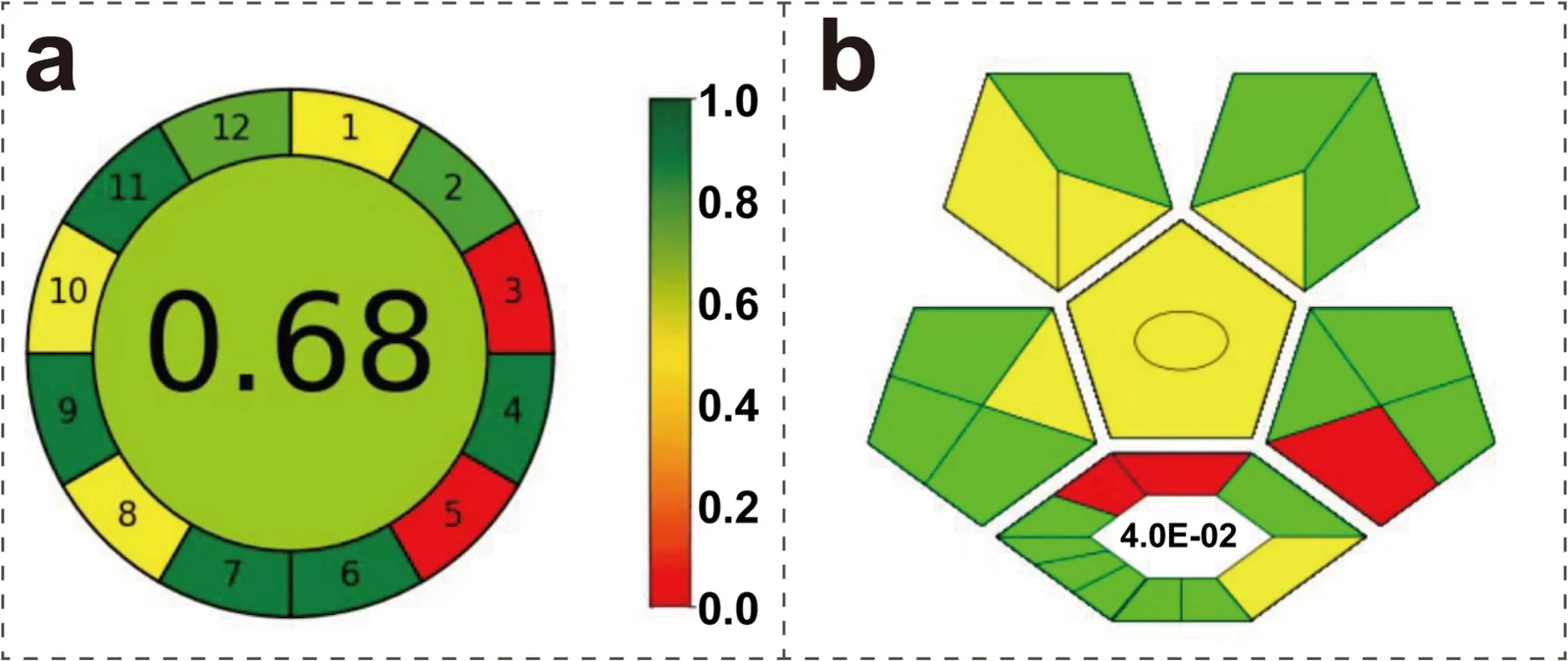
Method greenness assessment. (a) the AGREE assessment of NADES–SMIP–SPE strategy; (b) the GAPI assessment of NADES–SMIP–SPE strategy.

Compared with conventional methods such as maceration, reflux, and Soxhlet extraction, ultrasound-assisted NADES extraction offers significant advantages. Conventional methods rely on the natural diffusion of organic solvents and have difficulty disrupting cell walls, resulting in slow mass transfer, long extraction times, and low recovery, along with large solvent consumption, serious waste liquid pollution, and high energy consumption at elevated temperatures [69]. Ultrasonic cavitation can disrupt cell walls and enhance mass transfer; combined with green and natural NADES, it significantly improves extraction yield and recovery, substantially reduces extraction time, solvent consumption, and waste liquid discharge, and lowers energy consumption. Overall, this technology is efficient, time-saving, low-consumption, and green, showing broad application prospects [70]. In addition, AGREE and the Complex GAPI were used to comparatively evaluate the greenness of conventional extraction methods and the ultrasound-assisted NADES method. Fig. S8 **and** Fig. 11 show that the greenness of conventional extraction methods is lower than that of the ultrasound-assisted NADES method: the AGREE score of the ultrasound-assisted NADES method is 0.51, while conventional methods exhibit far more red indicators in the Complex GAPI assessment. Therefore, the ultrasound-assisted NADES method combines the advantages of high efficiency, energy saving, and environmental friendliness.

Although this study focused on the extraction and selective enrichment performance of the integrated platform, the recovery and reuse of NADES are of great significance for its practical sustainability. NADES systems possess extensive hydrogen-bond networks, and their recovery can be achieved by adding water to disrupt these hydrogen bonds, followed by purification or concentration through evaporation or other separation techniques [71]. The recovered NADES can potentially be reused in subsequent extraction cycles. However, this study did not systematically optimize the recovery and reuse of NADES. Future work will focus on establishing low-energy, high-recovery NADES recovery processes and evaluating recovery efficiency, reuse cycles, and the stability of extraction performance, thereby further supporting the scalable and sustainable application of this platform.

## 4 Conclusions

This study presents an integrated platform combining ultrasound-assisted NADES extraction with surface molecularly imprinted solid-phase extraction (SMIP-SPE) for the selective enrichment of flavonoid aglycones from safflower. The strategy couples green ultrasound–assisted NADES extraction with SMIP–SPE, thereby effectively overcoming the inherent selectivity limitation of NADES. Choline chloride–acetic acid NADES (molar ratio 1:2, 50% aqueous solution) was identified as the optimal green extractant under ultrasonic irradiation. Guided by DFT predictions and ML optimization, the SMIP prepared on SU–102 support exhibited a satisfactory adsorption capacity (44.639 mg/g) and high selectivity towards flavonoids. The SMIP–SPE module efficiently captured the target flavonoids from the complex extract, with the recovery remaining above 90% after seven cycles. Multiscale analyses, including DFT calculations and MD simulations, consistently revealed that hydrogen bonding serves as the key driving force throughout the separation process. The developed ultrasound-NADES-SMIP-SPE platform, with the aid of ultrasound, offers a green and efficient route for flavonoid recovery from safflower. By adjusting template molecules, this system can sustainably separate high-purity active ingredients from natural medicinal plants and is expected to become a candidate technology for green, scalable, and industrial separation of plant active ingredients.

## CRediT authorship contribution statement

**Zhiliu Liu:** Writing – original draft. **Kechun Wang:** Software. **Xiaoqing Zheng:** Methodology. **Xi Zhao:** Investigation, Formal analysis. **Yidan Jia:** Software. **Yan Wang:** Investigation. **Beibei Xiang:** Investigation. **Lijie Wu:** Writing – review & editing. **Xiaoxia Li:** Funding acquisition.

## Declaration of competing interest

The authors declare that they have no known competing financial interests or personal relationships that could have appeared to influence the work reported in this paper.

## Appendix A Supplementary data

Supplementary Data 1 The Supplementary material contains Figures S1–S8, Tables S1–S10 providing additional data supporting the results presented in the main text.

## References

[1] Ren F., Tan Z., Hu S., Rao C., Xiang Q., Wen J., Chen Y., Peng C. (2025). Pharmacological actions and applications of safflower flavonoids. Front. Nutr..

[2] Zhang B., Dong E., Liu L., Tian J., Chen S., Zhu L., Zhang J. (2025). Ten recommendations for the high-quality development of traditional chinese medicine in the new era. Acupunct. Herb. Med..

[3] Huang X., Jia A., Liu S., Zhao H., Ding C., Yan C. (2026). Extraction process and antioxidant and antimicrobial activities of total flavonoids from broussonetia papyrifera leaves. Sci. Rep..

[4] Huang J., Zhao Y., Wang M., Tian X., Cui D., Wang Q., Li X., Wu H., Yang W. (2025). Panax notoginseng polysaccharides: a review focusing on the extraction, isolation, structural characterization, biological activities and applications. Acupunct. Herb. Med..

[5] Fu X., Wang D., Belwal T., Xu Y., Li L., Luo Z. (2021). Sonication-synergistic natural deep eutectic solvent as a green and efficient approach for extraction of phenolic compounds from peels of carya cathayensis sarg. Food Chem..

[6] Li H., Du K., Liu C., Yang Y., Ye C., Li X., Chang Y. (2025). Enhancing*Verbena officinalis* l. Antioxidant Yield through Natural Deep Eutectic Solvents and MOF Synergetic Application, Green Chem..

[7] Yan Y., Shen L., Wang Y., Gong B., Li Z., Qiu H. (2025). Development of switchable deep eutectic solvents: applications in extraction of natural products. Chin. Chem. Lett..

[8] Li H., Yang K., Yang Y., Ding L., Li X. (2025). Natural deep eutectic solvents (NADES) in drug delivery systems: characteristics, applications, and future perspectives. Int. J. Pharm..

[9] Fu X., Wang D., Belwal T., Xie J., Xu Y., Li L., Zou L., Zhang L., Luo Z. (2021). Natural deep eutectic solvent enhanced pulse-ultrasonication assisted extraction as a multi-stability protective and efficient green strategy to extract anthocyanin from blueberry pomace. LWT.

[10] Huo H., Bao H., Liu T., Wu S., Yang B., Gao R., Song M., Kan H. (2026). Ultrasound-assisted extraction for enhanced recovery and bioactivity of polyphenols from tuber formosanum. Ultrason. Sonochem..

[11] Shi L., Wu Y.Y., Zhu Y.T., Yu T.S., Zhou Y., Xie M.D., Pang H.Q. (2025). The rise of green solvents: application and efficiency of deep eutectic solvents in the extraction of flavonoids. J. Food Sci..

[12] Cui J., Fang D., Tian X., Peng J., Chen D., Xu S., Ma L. (2023). Sustainable conversion of cottonseed hulls to valuable proanthocyanidins through ultrasound-assisted deep eutectic solvent extraction. Ultrason. Sonochem..

[13] Xu R., Chaudhary K., Altemimi A.B., Khalid S., Ansar S., Abid N., Abdi G., Aadil R.M. (2025). Ultrasound-assisted extraction with deep eutectic solvents for valorization of agro-food waste: a sustainable and eco-friendly approach. Ultrason. Sonochem..

[14] Shen L., Pang S., Zhong M., Sun Y., Qayum A., Liu Y., Rashid A., Xu B., Liang Q., Ma H., Ren X. (2023). A comprehensive review of ultrasonic assisted extraction (UAE) for bioactive components: principles, advantages, equipment, and combined technologies. Ultrason. Sonochem..

[15] Jacobson B., Li S., Daly P., Elgar C.E., Abbott A.P., Feeney A., Prentice P. (2024). Observation of cavitation dynamics in viscous deep eutectic solvents during power ultrasound sonication. Faraday Discuss..

[16] Wang C., You Y., Huang W., Zhan J. (2025). Efficient recovery of procyanidins from grape residue by UAE-NADES and macroporous resin. Ultrason. Sonochem..

[17] Yang T., Tan C., Wang R., Shi Y., Liu J., Liang Y., Ren C., Wang Y., Yao Y. (2026). Ultrasound-assisted hydrophobic deep eutectic solvent extraction and purification of ursolic acid and oleanolic acid from cornus officinalis: optimization and mechanistic insights. Ultrason. Sonochem..

[18] Singh I.P., Sharma A. (2026). Natural deep eutectic solvents as green extraction media for nutritional and functional bioactives. J. Agric. Food Chem..

[19] Ye K., Xu S., Zhou Q., Wang S., Xu Z., Liu Z. (2023). Advances in molecular imprinting technology for the extraction and detection of quercetin in plants. Polymers.

[20] Hou Y.J., Luo Y.Q., Li Q., Zhang H., Njolibimi M., Li W.J., Hong B., Zhao C.J. (2024). A new multi-template molecularly imprinted polymer for separation and purification of dioscin, protodioscin, and diosgenin from purple yam. Food Chem..

[21] Yang Y., Zhao X., Wang K., Liu J., Sun W., Du K., Row K.H., Li X. (2026). On-site screening from portability to intelligence: molecularly imprinted sensors for rapid early warning of mycotoxins. Trac-Trends Anal. Chem..

[22] Li H., Wang S., Ma J., He Z., Jia Y., Sun W., Han X. (2026). MOF-supported surface-imprinted polymer for hazard governance ofaristolochic acids in herbal matrices: A safety-control strategy supported bymultiscale simulations. J. Chromatogr. A.

[23] Li C.N., Tao Z.P., Liu L., Yuan D., Han Z.B. (2024). Cation exchange strategy of ellagic acid-based microporous metal-organic framework for efficient CO2/n2 and CO2/CH4 separation. Chem. Eng. J..

[24] Wisnuwardhani H.A., Ibrahim S., Mukti R.R., Damayanti S. (2024). Computational and experimental investigation on functional monomer selection for a novel molecularly imprinting polymer of remdesivir. J. Mol. Liq..

[25] Rafie S.F., Abu-Zahra N., Sabetvand R. (2025). From thin air to pure water: enhancing atmospheric water harvesting with molecular dynamics simulations of pristine and porous zirconium-based MOF-801. J. Mol. Liq..

[26] Fang Y., Zhang P., Wang S., Li L., Sheng Z. (2025). Ultrasonic-assisted extraction of luteolin from peanut shells using supramolecular solvents and its molecular mechanism. Ultrason. Sonochem..

[27] Zhang W., Chen R., Li J., Huang T., Wu B., Ma J., Wen Q., Tan J., Huang W. (2023). Synthesis optimization and adsorption modeling of biochar for pollutant removal via machine learning. Biochar.

[28] Peng Y., Du K., Yue H., Li H., Li H., Liu M., Shangguan S., He X., Li X., Chang Y. (2026). Integrated deep eutectic system enrichment and AI-assisted high-throughput visual detection for hg2+ in environmental samples. J. Adv. Res..

[29] Liao H., Yan Y., Yao Y., Duca G., Liu Y. (2026). Accelerating the discovery of single-atom catalysts for nitrate reduction via machine learning and DFT. Chin. Chem. Lett..

[30] Li H., Yue H., Li H., Zhu M., He X., Liu M., Li X., Qiu F. (2025). High-throughput point-of-care serum iron testing utilizing machine learning-assisted deep eutectic solvent fluorescence detection platform. J. Colloid Interface Sci..

[31] Pang B., Chen Y., Lyu H., Li C., Zhang L., Fan X., Zhao L., Tu J., Dong L., Zhang J. (2025). Modern technological advances in traditional medicine: global insights from the, top 50 high-impact researches. Acupunct. Herb. Med..

[32] Taoufik N., Boumya W., Elmoubarki R., Elhalil A., Achak M., Abdennouri M., Barka N. (2022). Experimental design, machine learning approaches for the optimization and modeling of caffeine adsorption. Mater. Today Chem..

[33] Liu C., Balasubramanian P., An J., Li F. (2025). Machine learning prediction of ammonia nitrogen adsorption on biochar with model evaluation and optimization. npj Clean Water.

[34] Zhang T., Doert T., Wang H., Zhang S., Ruck M. (2021). Inorganic synthesis based on reactions of ionic liquids and deep eutectic solvents. Angew. Chem.-Int. Edit..

[35] Svensson Grape E., Chacón-García A.J., Rojas S., Pérez Y., Jaworski A., Nero M., Åhlén M., Martínez-Ahumada E., Galetsa Feindt A.E., Pepillo M., Narongin-Fujikawa M., Ibarra I.A., Cheung O., Baresel C., Willhammar T., Horcajada P., Inge A.K. (2023). Removal of pharmaceutical pollutants from effluent by a plant-based metal–organic framework. Nature Water 1.

[36] Ghattas B., Manzon D. (2023). Machine learning alternatives to response surface models. Mathematics.

[37] Pei X., Pei J., Hou H., Zhao Y. (2025). Optimizing casting process using a combination of small data machine learning and phase-field simulations. npj Comput. Mater..

[38] Shishov A., Markova U., Mulloyarova V., Tolstoy P., Shkaeva N., Kosyakov D., Das N.K., Banerjee T. (2024). Deep eutectic solvent as stationary phase for flow analysis: automated trace metal determination in food products. Anal. Chim. Acta.

[39] Wang J., Zhou L., Wang Z., Tao Y., Chen L., Zhang W. (2023). Effects of natural deep eutectic solvent on the extraction efficiency and antioxidant activities of toona sinensis seed polyphenols: composition and mechanism. Food Biosci..

[40] Hikmawanti N.P.E., Ramadon D., Jantan I., Mun Im A. (2021). Natural deep eutectic solvents (NADES): phytochemical extraction performance enhancer for pharmaceutical and nutraceutical product development. Plants.

[41] Alshammari O.A.O., Almulgabsagher G.A.A., Ryder K.S., Abbott A.P. (2021). Effect of solute polarity on extraction efficiency using deep eutectic solvents. Green Chem..

[42] Chevé-Kools E., Choi Y.H., Roullier C., Ruprich-Robert G., Grougnet R., Chapeland-Leclerc F., Hollmann F. (2025). Natural deep eutectic solvents (NaDES): green solvents for pharmaceutical applications and beyond. Green Chem..

[43] Alfaleh A.A., Sindi H.A. (2024). Systematic study on date palm seeds (phoenix dactylifera l.) Extraction optimisation using natural deep eutectic solvents and ultrasound technique. Sci. Rep..

[44] Haider W., Lin Z., Gao K., Li J., Li Y., Zhong J., Yuan L., Chen Z., Chen Y., Ding Z., Zhou F., Zhou M. (2026). Ultrasound-assisted deep eutectic solvent extraction of polysaccharides from melastoma dodecandrum: RSM optimization, ANN prediction, kinetics, structural characterization and cellular antioxidant activity. Ultrason. Sonochem..

[45] Dzah C.S., Duan Y., Zhang H., Wen C., Zhang J., Chen G., Ma H. (2020). The effects of ultrasound assisted extraction on yield, antioxidant, anticancer and antimicrobial activity of polyphenol extracts: a review. Food Biosci..

[46] Lin S., Zhang H., Simal-Gandara J., Cheng K.W., Wang M., Cao H., Xiao J. (2022). Investigation of new products of quercetin formed in boiling water via UPLC-q-TOF-MS-MS analysis. Food Chem..

[47] Qiao L., Sun Y., Chen R., Fu Y., Zhang W., Li X., Chen J., Shen Y., Ye X. (2014). Sonochemical effects on 14 flavonoids common in citrus: relation to stability. PLoS One.

[48] He X., Yang Y., Cao Y., Wang Z., Li H., Liu Z., Wang Y., Li F., Zhang Q., Yusuf A., Li F., Hu M., Deng Y., Wang X., Liu Z., Sun W., Wang H., Li X. (2026). Poly(deep eutectic solvent)@MOF-801 for technical surveillance of carbaryl: pesticide residue safety control strategy. J. Environ. Manage..

[49] Huang H., Guo S., Xu Y., Ettoumi F.T.M.E., Fang J., Yan X., Xie Z., Luo Z., Cheng K. (2024). Valorization and protection of anthocyanins from strawberries (fragaria×ananassa duch.) By acidified natural deep eutectic solvent based on intermolecular interaction. Food Chem..

[50] Huang H., Zhu Y., Fu X., Zou Y., Li Q., Luo Z. (2022). Integrated natural deep eutectic solvent and pulse-ultrasonication for efficient extraction of crocins from gardenia fruits (gardenia jasminoides ellis) and its bioactivities. Food Chem..

[51] Yuan J., Gao Y., Tian X., Su W., Su Y., Niu S., Meng X., Jia T., Yin R., Hu J. (2024). Computational and experimental comparison of molecularly imprinted polymers prepared by different functional monomers—quantitative parameters defined based on molecular dynamics simulation. Molecules.

[52] Wang L., Li M., Wang Y., Xu L., He X., Li H., Li X. (2024). Harnessing two-dimensional magnetic poly(deep eutectic solvents) for matrix solid-phase dispersion extraction of polyphenols from roselle: promoting antiphotoaging strategy. Chem. Eng. J..

[53] Xu L., Zhang X., Yang W., Li H., Wang J., Wang L., He X., Wang Y., Li X. (2024). Advanced technology based on poly(deep eutectic solvent) core–shell nanomaterials enriched with fructus choerospondias phenols for efficient defense against UVB-induced ferroptosis. Chem. Eng. J..

[54] Han X., Wang S., Cui T., Yang Y., Li H., Zhang H., Zhou H., Li X. (2025). Sustainable defect-engineered imprinted UiO-66-NH_2_ for selective removal of aristolochic acids from medicinal herbs. ACS Sustain. Chem. Eng..

[55] Liu M., Xiang B., Li H., He X., Li H., Du K., Li X. (2025). A sustainable poly (deep eutectic solvents) based molecular imprinting strategy with experimental and theoretical elucidation: application for removal of atrazine in agricultural wastewater. Sep. Purif. Technol..

[56] Yang Y., Du K., Liu M., He X., Li H., Li H., Li X. (2025). A computer-assisted design of poly(deep eutectic solvent)@MIL-101-NH2(cr) imprinting strategy: selective removal of zearalenone from coix seeds. Food Chem..

[57] Wang T., Li G., Xue B., Guo P., Zhao X., Hao T., Liu D. (2025). A robust anionic metal–organic framework for efficient capture of rubidium: performance and mechanism study. Ind. Eng. Chem. Res..

[58] Chang Y., Zhou D., Kong X., Xu Z., Wu X., Xu A., Wang Y., Huang L., Wu D., Ban X., Huang H. (2026). Tailoring oxygen vacancies of bio-based metal-organic frameworks via biogenic gallate modulator for enhanced formaldehyde adsorption. J. Environ. Chem. Eng..

[59] Jomardani F., Shakeri R., Akbarzadeh R., Kokhdan S.N. (2026). Adsorption kinetics and isotherms of malachite green removal from aqueous solution using TiO2 loaded on f-MWCNTs nanocomposite. Sci. Rep..

[60] Usevičiūtė L., Kaklauskas A.R., Danila V., Januševičius T. (2025). Enhanced aqueous phosphate removal using chitosan-modified zirconium-loaded cork biochar. Sci. Rep..

[61] Peng X., Yang P., Dai K., Chen Y., Chen X., Zhuang W., Ying H., Wu J. (2020). Synthesis, adsorption and molecular simulation study of methylamine-modified hyper-cross-linked resins for efficient removal of citric acid from aqueous solution. Sci. Rep..

[62] Guan S., Wang Y., Hu T., Che L., Wang X., Huang Y., Xia Z. (2024). Study on the selectivity of molecular imprinting materials determined through hydrogen bonding on template molecular structures of flavonoids. Molecules.

[63] Wang H., Wang H., Cheng L., Li R., Yang T., Wang W., Ma J., Sun Z. (2024). Hydrogen bonds-based flexible regulation of acetaminophen adsorption/desorption behavior in aqueous system: role of the smart thermoresponsive graphene oxide. Chem. Eng. J..

[64] Jabbari A., Jabbari M., Zare E.N. (2026). Synthesis and evaluation of a novel MOF/polymer nanohybrid for ph-sensitive adsorption of two natural products with similar structure. Talanta.

[65] Iranzad R., Liu X. (2025). A review of random forest-based feature selection methods for data science education and applications. Int. j, Data Sci. Anal..

[66] Jia Y., Liu Y., Li H., Shangguan S., Wang K., Han X., Li X. (2026). Systematic optimization of graphene oxide-enhanced flavonoid liberation from resina draconis: decoding the underlying molecular mechanisms. Microchem. J..

[67] Płotka-Wasylka J., Wojnowski W. (2021). Complementary green analytical procedure index (ComplexGAPI) and software. Green Chem..

[68] Dong Y., Kong D., Li X., Li H., Chen M., Dou Z., Li X., Wang H. (2025). Application of a novel deep eutectic solvent as an eco-friendly medium for green extraction of cadmium from herbal medicines. Sep. Purif. Technol..

[69] Cannavacciuolo C., Pagliari S., Celano R., Campone L., Rastrelli L. (2024). Critical analysis of green extraction techniques used for botanicals: trends, priorities, and optimization strategies-a review. TrAC Trends Anal. Chem..

[70] Boateng I.D., Clark K. (2024). Trends in extracting agro-byproducts’ phenolics using non-thermal technologies and their combinative effect: mechanisms, potentials, drawbacks, and safety evaluation. Food Chem..

[71] Huang H., Ying P., Wang Y., Wu Q., Wang L., Fu X. (2025). Temperature-dependent convection induced incremental extraction of anthocyanins from melastoma dodecandrum lour. Based on Recyclable Natural Deep Eutectic System, Food Chem..

