# Supplementary material for: Natural deep eutectic solvent ultrasound extraction and imprinting recognition for sustainable harvesting of flavonoid aglycones from safflower: Experimental and multi-scale simulations
Source: Ultrason Sonochem. 2026 Oct 22;134:108128. doi: 10.1016/j.ultsonch.2026.108128 (PMC13740884; doi:10.1016/j.ultsonch.2026.108128)
Supplement: Supplementary file 1 [file mmc1.docx]

**Natural deep eutectic solvent ultrasound extraction and imprinting recognition for sustainable harvesting of flavonoid aglycones from safflower: experimental and multi-scale simulations**

Zhiliu Liu ^a,b,c^, Kechun Wang ^a,b,c,#^, Xiaoqing Zheng ^e,#^, Xi Zhao ^a,b,c^,Yidan Jia ^a,b,c^, Yan Wang ^e^，Beibei Xiang ^b,c*^, Lijie Wu ^a*^, Xiaoxia Li ^a,b,c,d*^

*^a^ School of Chinese Materia Medica, Tianjin University of Traditional Chinese Medicine, Tianjin, 301617, China*

*^b^ State Key Laboratory of Chinese Medicine Modernization, Tianjin University of Traditional Chinese Medicine, Tianjin, 301617, China*

*^c^ Tianjin Key Laboratory of Therapeutic Substance of Traditional Chinese Medicine, Tianjin University of Traditional Chinese Medicine, Tianjin, 301617, China*

*^d^ China-Ghana Belt and Road Joint Laboratory on Traditional Medicine, Tianjin University of Traditional Chinese Medicine, Tianjin, 301617, China*

*^e^ School of Chinese Medicine and Pharmaceutical Engineering, Tianjin University of Traditional Chinese Medicine, Tianjin, 301617, China*

\# The authors contributed equally to the first author.

^[1]^

1. **Ultrasonic power calibration method**

Using 30 g of deionized water as the medium, the temperature rise curve during ultrasonic irradiation was recorded. The calibration was performed in triplicate. The actual acoustic power was calculated according to the equation:

$P$ = $m$ × $C_{P}$ × $(\frac{dT}{dt})$	(1)

where P is the acoustic power (W), m is the mass of water (g), and Cp denotes the specific heat capacity of water, approximately 4.18 J/g°C.

2. **Controlled UAE conditions: temperature regulation and positional standardization**

An external circulating cooling system was connected to the ultrasonic water bath to maintain the set extraction temperature throughout the 30 min sonication. The circulating cooling water actively removed the heat generated by the transducer, keeping the water bath temperature stable at the set value. The extraction vessel was placed in the water bath to ensure that the set extraction temperature was maintained during the entire 30 min sonication. In each experiment, a calibrated thermometer was used to monitor the actual temperature inside the extraction vessel in real time, and temperature changes were recorded, with fluctuations controlled within ±1 °C. All extraction kinetics experiments were performed under these controlled temperature conditions, ensuring good comparability, reproducibility, and reliability of the data obtained at different temperatures and time points.

Ultrasound-assisted extraction experiments were carried out using Falcon centrifuge tubes as extraction vessels. All tubes were secured in a dedicated rack precisely matched to the built-in slots of the ultrasonic water bath, ensuring that each sample tube occupied an identical spatial position within the bath and avoiding positional deviations among parallel samples. In all extraction batches, the water level in the ultrasonic water bath was maintained at a constant level, 5 cm above the top of the Falcon centrifuge tubes, and the liquid volume in each Falcon centrifuge tube was kept uniform.

**Fig. S1.** Ultrasonic power calibration chart

**Fig. S2.** IGMH scatter plots and rainbow-colored δg^inter^ isosurfaces of quercetin polymers with different ratios (SMD).

**Fig. S3.** IGMH scatter plots and rainbow-colored δg^inter^ isosurfaces of quercetin polymers with different ratios.

**Fig. S4.** Evaluation of SMIP adsorption capacity in mixed standard solution

**Fig. S5.** Scatter plot of actual values - predicted values (training set).

**Fig. S6.** Scatter plot of actual values - predicted values (test set).

**Fig. S7.** (a-b) TEM of SU-102; (c-d) TEM of SMIP without acid washing; (e-f) TEM of SMIP after acid washing.

**Fig. S8.** (a) the AGREE assessment of traditional extraction strategy; (b) the GAPI assessment of traditional extraction strategy.

**Table S1** Ultrasonic power determined by disequilibrium calorimetry.

| Power | P (W) | Sound power density (W/cm³) |
| --- | --- | --- |
| **Mean** | 2.12 | 0.071 |
| **SD** | 0.18 | 0.006 |
| **RSD (%)** | 8.49 | 8.45 |

**Table S2** ESP parameters of various molecules.

| Molecules | ChCl | Acetic acid | NADES | Quercetin | MAA |
| --- | --- | --- | --- | --- | --- |
| Color scale data range (a.u.) | -0.11 | -0,05 | -0.09 | -0.08 | -0.05 |
|  | 0.10 | 0.08 | 0.09 | 0.11 | 0.08 |

**Table S3** HPLC system suitability.

| Repeatability (n=6) | Precision (n=6) | USP tailing factor | Symmetry factor | Number of theoretical plates | LOD  (mg/L) |
| --- | --- | --- | --- | --- | --- |
| RSD (%) | |  |  |  |  |
| Peak area | Peak area |  |  |  |  |
| 0.760 | 1.011 | 1.138 | 1.1280 | 12746.530 | 0.700 |

**Table S4** Viscosity test data at different temperatures

| Number | Viscosity (mPa·s) | Viscosity (mPa·s) | Viscosity (mPa·s) | Viscosity (mPa·s) | Viscosity (mPa·s) |
| --- | --- | --- | --- | --- | --- |
|  | 20℃ | 30℃ | 40℃ | 50℃ | 60℃ |
| II | 5.1 | 2.5 | 2.3 | 2.2 | 1.9 |
| III | 13.3 | 5.5 | 5.0 | 4.9 | 4.5 |
| IV | 14.6 | 12.1 | 8.8 | 6.0 | 5.6 |
| V | 3.8 | 2.0 | 1.8 | 1.4 | 1.1 |
| VI | 30.4 | 24.8 | 10.6 | 8.8 | 7.9 |
| VII | 15.3 | 15.0 | 12.1 | 10.2 | 9.3 |
| VIII | 46.2 | 41.8 | 26.3 | 24.9 | 20.2 |
| IX | 3.7 | 3.5 | 2.8 | 2.3 | 1.9 |

**Table S5** Viscosity test data of NADES at different ultrasound power settings

| ChCl: Acetic acid | 50 W | 60 W | 70 W | 80 W | 90 W | 100 W |
| --- | --- | --- | --- | --- | --- | --- |
| Viscosity (mPa·s) | 4.8 | 4.5 | 4.1 | 3.4 | 2.9 | 2.1 |

**Table S6** Gibbs free energy (ΔG) of polymers obtained from theoretical calculations.

| Materials | 1:1 | 1:2 | 1:3 | 1:4 |
| --- | --- | --- | --- | --- |
| ΔG (kcal/mol) | -2.783 | -4.971 | -7.871 | -20.594 |

**Table S7** Gibbs free energy (ΔG) of polymers obtained from theoretical calculations (SMD).

| Materials | 1:1 | 1:2 | 1:3 | 1:4 |
| --- | --- | --- | --- | --- |
| ΔG (kcal/mol) | 2.019 | 2.274 | 0.897 | -1.359 |

**Table S8** BET specific surface area and particle size analysis.

| Parameter | SU-102 | SMIP | MIP |
| --- | --- | --- | --- |
| BET surface area (m^2^/g) | 135.580 | 18.103 | 191.259 |
| Average pore size (nm) | 14.132 | 11.423 | 3.498 |
| Porous volume (cm^3^/g) | 0.365 | 0.032 | 0.137 |

**Table S9** Adsorption capacity of SMIP for various substances in mixed standard solution

| Analyte | Adsorption capacity_1_ (mg/g) | Adsorption capacity_2_ (mg/g) | Adsorption capacity_3_ (mg/g) | Average (mg/g) |
| --- | --- | --- | --- | --- |
| Protocatechuic acid | 0.095 | 0.155 | 0.007 | 0.086 |
| Syringic acid | 0.360 | 0.423 | 0.305 | 0.363 |
| Ferulic acid | 0.385 | 0.504 | 0.334 | 0.4075 |
| Luteolin | 4.847 | 5.724 | 4.285 | 4.952 |
| Quercetin | 6.223 | 7.047 | 5.767 | 6.345 |
| Apigenin | 3.373 | 2.874 | 2.874 | 3.041 |
| Kaempferol | 3.897 | 4.138 | 3.579 | 3.871 |

**Table S10** Data of Reusability experiment.

| Recycles | Extraction rate (%) | Extraction rate (%) | Extraction rate (%) | Average extraction rate (%) |
| --- | --- | --- | --- | --- |
| 1 | 93.54 | 93.47 | 93.49 | 93.50 |
| 2 | 93.32 | 93.42 | 93.32 | 93.35 |
| 3 | 92.62 | 92.76 | 92.51 | 92.63 |
| 4 | 92.03 | 92.16 | 92.03 | 92.07 |
| 5 | 91.45 | 91.89 | 91.71 | 91.68 |
| 6 | 90.69 | 90.83 | 90.73 | 90.75 |
| 7 | 90.54 | 90.35 | 90.27 | 90.38 |

1. ^*^ **Corresponding author.**

   *E-mail:* [lixx@tjutcm.edu.cn](mailto:lixx@tjutcm.edu.cn) (X. Li), [wulj0816@163.com](mailto:wulj0816@163.com) (L. Wu), xiangbeibei03230@163.com (B. Xiang).
